# Lachnospiraceae in the Food–Gut Axis: A Critical Review of Dietary Modulation, Immune Regulation, and Translational Barriers

**DOI:** 10.1111/1541-4337.70655

**Published:** 2026-09-28

**Authors:** Ruijun Wang, Zhiqi Li, Lina Zhang, Yuanming Pan, Zhanbiao He

**Affiliations:** ^1^ Department of Neurosurgery The Affiliated Hospital of Inner Mongolia Medical University Hohhot Inner Mongolia Autonomous Region China; ^2^ Cancer Research Center, Beijing Chest Hospital Capital Medical University Beijing China

**Keywords:** critical review, dietary fiber, food‐gut axis, immune regulation, Lachnospiraceae, short‐chain fatty acids, synbiotic formulations, translational barriers

## Abstract

The interplay between diet, gut microbiota, and host immunity is central to food science. The Lachnospiraceae family ferments dietary fibers to produce short‐chain fatty acids (SCFAs), notably butyrate and propionate, with immunomodulatory potential. This critical review distinguishes established findings from hypotheses. Strain‐level functional heterogeneity is substantial; findings from one genus cannot be generalized. Specific substrates (inulin, pectin, resistant starch, and arabinoxylan) and food processing methods shape SCFA profiles. Mechanistically, SCFAs regulate immunity via histone deacetylase inhibition, G‐protein‐coupled receptor activation, and Treg/Th17 modulation. However, most mechanistic data derive from rodent models; human evidence remains largely correlational and cross‐sectional. Three major knowledge gaps exist: the lack of strain‐level functional characterization, the absence of causal human intervention trials for the vast majority of Lachnospiraceae strains (with *A*naerobutyricum *soehngenii* being the sole exception, tested only in metabolic disease), and limited translation into food systems. Regulatory barriers (generally recognized as safe [GRAS] status exists only for *A. soehngenii* CH106 and strictly for food use; no other strain has GRAS or qualified presumption of safety [QPS]), safety concerns (rare opportunistic pathogenicity), and technological challenges (oxygen sensitivity and viability in food matrices) further hinder development. Postbiotics (preparations of inactivated microbial cells or their components) and metabolite‐based preparations (e.g., purified SCFAs or fermentation supernatants), alongside synbiotic formulations, offer practical near‐term alternatives. Importantly, under the International Scientific Association for Probiotics and Prebiotics (ISAPP) definition, purified metabolites without cellular biomass do not qualify as postbiotics. We conclude that although Lachnospiraceae hold promise for immunomodulatory functional foods, current evidence does not support clinical or commercial claims. Priority research directions include longitudinal human cohorts, strain‐level culturomics, and multi‐omics integration. This review provides a realistic, food science–centered framework for future investigations.

**Abbreviations**
3‐oxoLCA: 3‐oxolithocholic acid
5β‐R: 5β‐reductase
7α‐HSDH: 7α‐hydroxysteroid dehydrogenase
ACR50: American College of Rheumatology 50% improvement criteria
AhR: aryl hydrocarbon receptor
AMPK: AMP‐activated protein kinase
BLA: biologics license application
CAZymes: carbohydrate‐active enzymes
CD: Crohn's disease
CIA: collagen‐induced arthritis
CNKI: China National Knowledge Infrastructure
CRP: C‐reactive protein
CTL: cytotoxic T lymphocyte
CX3CR1: fractalkine receptor
DC: dendritic cell
DCA: deoxycholic acid
EFSA: European Food Safety Authority
EMA: European Medicines Agency
EPS: extracellular polysaccharides
EU: European Union
FDA: Food and Drug Administration
FMT: fecal microbiota transplantation
FOS: fructo‐oligosaccharides
FXR: farnesoid X receptor
GDM: gestational diabetes mellitus
GMP: good manufacturing practice
GPCR: G‐protein‐coupled receptor
GPR109A: G‐protein‐coupled receptor 109A
GPR41: G‐protein‐coupled receptor 41
GPR43: G‐protein‐coupled receptor 43
GRAS: generally recognized as safe
GRN: GRAS Notice
GTDB: genome taxonomy database
HbA1c: glycated hemoglobin
HDAC: histone deacetylase
HLA: human leukocyte antigen
HOMA‐IR: homeostatic model assessment of insulin resistance
IBD: inflammatory bowel disease
ICI: immune checkpoint inhibitor
IL: interleukin
IND: investigational new drug
ISAPP: International Scientific Association for Probiotics and Prebiotics
isoalloLCA: isoallolithocholic acid
LBP: live biotherapeutic product
LPSN: List of Prokaryotic names with Standing in Nomenclature
MAA: marketing authorization application
MAMP: microbe‐associated molecular pattern
MAPK: mitogen‐activated protein kinase
MMS: microbiome‐modulating strategies
mTORC1: mechanistic target of rapamycin complex 1
NF‐κB: nuclear factor kappa‐light‐chain‐enhancer of activated B cells
NOD2: nucleotide‐binding oligomerization domain‐containing protein 2
NSCLC: non‐small cell lung cancer
ORR: objective response rate
PBMC: peripheral blood mononuclear cell
PD‐1: programmed cell death protein 1
PD‐L1: programmed death‐ligand 1
PFS: progression‐free survival
QPS: qualified presumption of safety
RA: rheumatoid arthritis
RCT: randomized controlled trial
RIN: Regulation Identification Number
ROS: reactive oxygen species
RS: resistant starch
SCFA: short‐chain fatty acid
SNP: single nucleotide polymorphism
STZ: streptozotocin
T2D: Type 2 diabetes
TFR: follicular regulatory T cell
TGF‐β: transforming growth factor beta
Th: T helper cell
TLR5: toll‐like receptor 5
TNF‐α: tumor necrosis factor alpha
Treg: regulatory T cell
US: United States
ZO‐1: zonula occludens‐1

## 1 Introduction and Methodology

### 1.1 Literature Search Strategy: A Critical Narrative Review

To ensure comprehensive coverage of the literature on Lachnospiraceae and their roles in immune regulation, host metabolism, and functional food applications, we performed a structured literature search across multiple electronic databases. The primary databases included PubMed/MEDLINE (for biomedical and microbiological literature), Web of Science Core Collection (for interdisciplinary coverage), Scopus (for broad scientific literature), and Google Scholar (for supplementary and grey literature). Additionally, we searched Chinese databases (China National Knowledge Infrastructure [CNKI] and Wanfang Data) to capture region‐specific studies and ClinicalTrials.gov to identify ongoing or completed clinical trials. The search period covered January 2000 to March 2026, reflecting the timeframe during which high‐throughput sequencing and microbiome research became widely available.

A complete search string for PubMed, including filters and MeSH terms, is provided; search strategies for other databases were adapted accordingly. The search strategy employed Boolean combinations of the following key terms: primary taxonomic terms (“Lachnospiraceae,” “*Roseburia*,” “*Blautia*,” “*Eubacterium rectale*,” and “*Coprococcus*”) were combined with immune‐related terms (“immune regulation,” “immunomodulation,” “treg cells,” “Th17 cells,” “regulatory T cells,” and “immune tolerance”), metabolic terms (“short‐chain fatty acids,” “SCFAs,” “butyrate,” “propionate,” and “acetate”), and food‐relevant terms (“dietary fiber,” “prebiotics,” “functional foods,” “gut microbiota,” “intestinal barrier,” and “tight junction proteins”). For translational aspects, we included terms such as “therapeutic applications,” “cancer immunotherapy,” “autoimmune diseases,” and “metabolic diseases.”

This review is a critical narrative review, not a formal systematic review. Therefore, we did not perform a PRISMA flow diagram, a formal risk of bias assessment, or a quantitative meta‐analysis. However, to enhance transparency and reproducibility, we applied predefined inclusion and exclusion criteria. The initial search across all databases yielded 1247 records after the preliminary retrieval. After automated and manual deduplication (*n* = 286 removed), 961 records remained for title and abstract screening. Of these, 774 records were excluded as they were clearly irrelevant to the scope of this review (e.g., studies on environmental Lachnospiraceae not associated with human hosts or articles lacking any dietary or immunological context), leaving 187 full‐text articles for detailed assessment. Of these 187 articles, 44 were excluded for the following reasons: lack of primary data (*n* = 12), no relevant immune‐ or dietary‐related outcomes (*n* = 18), duplicate publications not detected earlier (*n* = 6), and studies with methodological descriptions insufficient for critical evaluation (*n* = 8). Ultimately, 143 studies were included in this review, comprising 82 preclinical studies (in vitro and animal models), 46 human observational studies (cross‐sectional and cohort), and 15 interventional trials (including randomized controlled trials (RCTs) and open‐label studies). Inclusion criteria were (i) peer‐reviewed original research articles, systematic reviews, or meta‐analyses; (ii) studies directly investigating Lachnospiraceae in relation to host immunity, metabolism, or food components; (iii) preclinical studies (in vitro, ex vivo, or animal models) as well as human studies (observational or interventional); and (iv) publications in English or Chinese. Exclusion criteria were conference abstracts without full‐text availability, studies lacking primary data or rigorous methodological descriptions, duplicate publications, and studies that did not address immune‐relevant or food‐relevant outcomes.

Data were extracted thematically using a standardized form that captured (i) study design; (ii) Lachnospiraceae taxa (at family, genus, species, or strain level); (iii) methodological approaches (16S rRNA sequencing, metagenomics, metabolomics, and culture‐based methods); (iv) immune parameters measured (cytokine profiles, immune cell subsets, and antibody responses); (v) metabolic outputs (short‐chain fatty acid [SCFA] quantification and bile acid profiling); and (vi) clinical outcomes (disease severity and treatment response). This thematic extraction was designed to support critical synthesis rather than quantitative aggregation. To assist readers in interpreting the weight of the evidence presented throughout this review, we categorized the strength of evidence for each major finding into four levels based on predefined criteria (Table 1).

**TABLE 1:** Criteria for grading evidence strength based on study design and sample size.

Evidence level	Definition	Study design requirements
**Strong**	Consistent findings from multiple well‐designed studies with large sample sizes	≥2 large‐scale RCTs (*n* ≥ 100 per arm) or multiple consistent meta‐analyses with low heterogeneity
**Moderate**	Findings from well‐designed observational studies or small‐scale RCTs	Well‐designed cohort studies (*n* ≥ 200), case‐control studies, or small‐scale RCTs (*n* = 30–99 per arm) with consistent results
**Low**	Findings from uncontrolled studies, case series, or mechanistic studies primarily from animal models	Uncontrolled observational studies, case series (*n* ≥ 10), or animal model studies with clear translational relevance
**Very low**	Findings from expert opinions, case reports, or preliminary in vitro studies only	Case reports (*n* < 10), expert opinions, or in vitro studies without in vivo validation

Abbreviation: RCT, randomized controlled trial.

These designations are explicitly indicated in the discussion of each thematic section to provide readers with a clear assessment of the evidentiary basis for each claim. We explicitly acknowledge that the absence of formal quality assessment tools (e.g., QUADAS‐2 and SYRCLE's RoB) is an inherent limitation of the narrative approach, and we have highlighted this limitation in Section 6. Nevertheless, by critically evaluating evidence strength, discussing conflicting findings, and distinguishing between preclinical and clinical data, we aimed to provide a balanced and rigorous synthesis.

Two authors (initials blinded) independently screened titles and abstracts. The inter‐reviewer agreement rate was 94.6%; disagreements (*n* = 52) were resolved through discussion or consultation with a third reviewer. Full texts of potentially eligible articles were retrieved and assessed. Disagreements were resolved through discussion or consultation with a third reviewer. Data were extracted thematically using a standardized form that captured study design, Lachnospiraceae taxa (at family, genus, species, or strain level), methodological approaches (16S rRNA sequencing, metagenomics, metabolomics, and culture‐based methods), immune parameters measured (cytokine profiles, immune cell subsets, and antibody responses), metabolic outputs (SCFA quantification and bile acid profiling), and clinical outcomes (disease severity and treatment response). This thematic extraction was designed to support critical synthesis rather than quantitative aggregation. To assist readers in interpreting the weight of the evidence presented throughout this review, we categorized the strength of evidence for each major finding into four levels—strong, moderate, low, and very low—based on study design, sample size, and consistency of results. These designations are explicitly indicated in the discussion of each thematic section. We explicitly acknowledge that the absence of formal quality assessment is a limitation of the narrative approach, and we have highlighted this limitation in the discussion (Section 6). Nevertheless, by critically evaluating evidence strength, discussing conflicting findings, and distinguishing between preclinical and clinical data, we aimed to provide a balanced and rigorous synthesis.

### 1.2 High Abundance, Diversity, and Ecological Importance of Lachnospiraceae

The gut microbiota is now recognized as a central regulator of host energy metabolism, nutrient absorption, neuroactive potential, immune development, and even gut‐brain signaling (Flint et al. 2012; Valdes et al. 2018; Valles‐Colomer et al. 2019; Cryan et al. 2020; Notting et al. 2023; Jiang, Huang, et al. 2025). Within this complex microbial ecosystem, the Lachnospiraceae family (phylum Firmicutes, class Clostridia, order Clostridiales) stands out as one of the most consistently prevalent and abundant bacterial groups in the intestines of healthy individuals (Zhao et al. 2026). Metagenomic studies across diverse human populations have reported that Lachnospiraceae typically constitute 15%–25% of the total gut microbiota, and their abundance is positively correlated with long‐term dietary fiber intake (Meehan and Beiko 2014; Zaplana et al. 2024).

The family exhibits substantial phylogenetic diversity. As of 2025, the genome taxonomy database (GTDB) recognizes over 60 genera and more than 387 species‐level phylogroups within Lachnospiraceae, although many remain uncultured (Sorbara et al. 2020). Common genera found in human fecal samples include Roseburia, Lachnospira, Coprococcus, Blautia, Eubacterium, Dorea, Agathobacter, and Anaerostipes. Importantly, these genera display marked functional differentiation at the species and even strain level (Vacca et al. 2020) (Figure 1). For example, although some species are potent butyrate producers, others preferentially produce propionate or acetate, and some possess mucin‐degrading capabilities that allow them to colonize the mucus layer (Singh et al. 2023).

**FIGURE 1:**
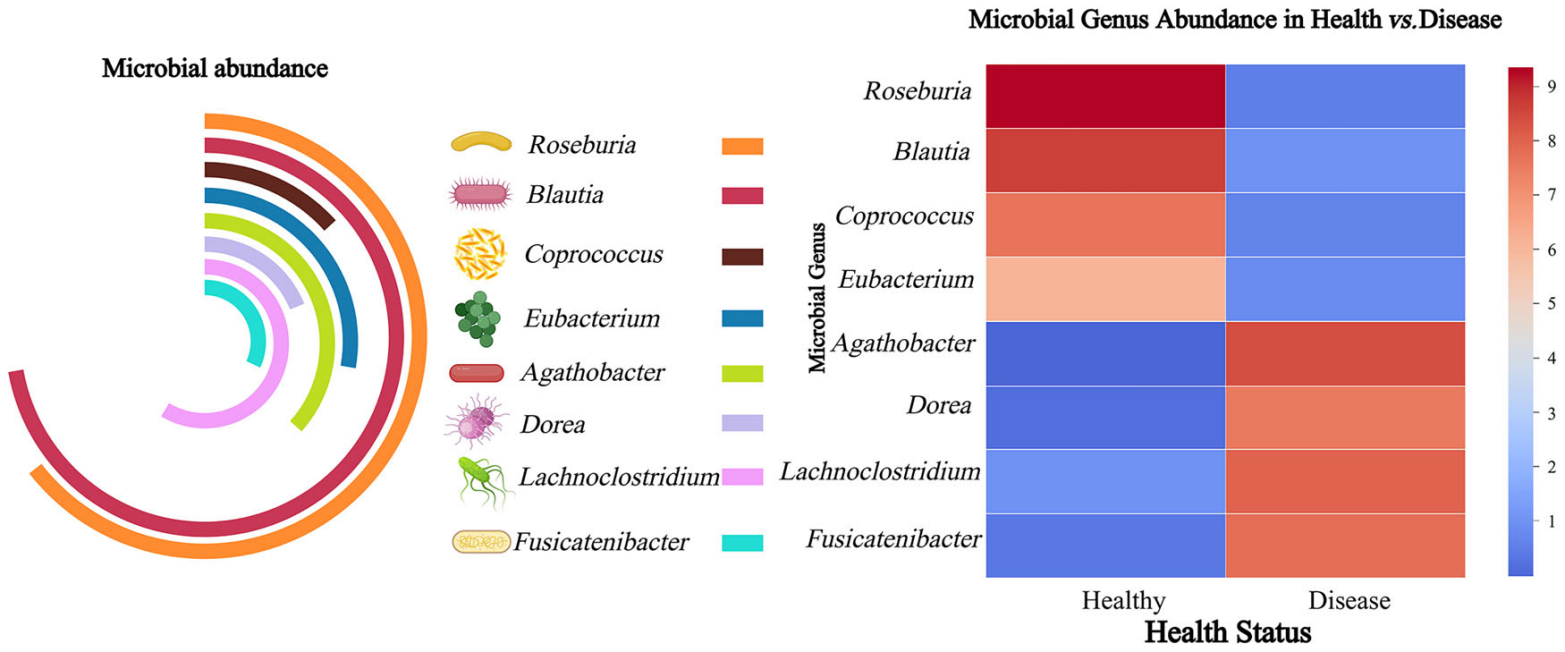
Microbial genus abundance and comparative analysis between healthy and disease states. (Left) Relative abundance profile of the selected gut microbial genera. The concentric curved bars represent the abundance levels of specific genera, including Roseburia, Blautia, Coprococcus, Eubacterium, Agathobacter, Dorea, Lachnoclostridium, and Fusicatenibacter. (Right) Heatmap displaying the abundance variations of the corresponding microbial genera between the “Healthy” and “Disease” groups. The color scale transitions from blue (low relative abundance) to red (high relative abundance). The data reveals that genera such as Roseburia, Blautia, and Coprococcus are predominantly enriched in the healthy group, whereas genera, including Dorea, Lachnoclostridium, and Fusicatenibacter, show marked enrichment in the disease group.

As anaerobic fermenters, Lachnospiraceae metabolize undigested dietary polysaccharides in the low‐oxygen environment of the distal gut. Their primary metabolic outputs are SCFAs, including butyrate, propionate, and acetate. These SCFAs are not merely waste products; they serve essential signaling and nutritional functions for the host. Butyrate, for instance, is the preferred energy source for colonocytes and reinforces the intestinal barrier by upregulating tight junction proteins (e.g., zonula occludens‐1 (ZO‐1), occludin) and inducing mucin secretion (Donohoe et al. 2011). It also limits pathogen colonization by lowering luminal pH and by direct antimicrobial effects (Louis and Flint 2017).

Notably, some butyrate‐producing species, such as E. rectale and Roseburia intestinalis, also synthesize glutathione, a major cellular antioxidant (Lu et al. 2022; Fusco et al. 2023). By secreting glutathione or its precursors, these bacteria can reduce oxidative stress in intestinal epithelial cells, suppress inflammatory gene expression, promote epithelial repair, and inhibit carcinogenesis (Lu et al. 2022; Richie et al. 2024; Gao et al. 2026). Other Lachnospiraceae contribute to the conversion of primary bile acids (e.g., cholic acid and chenodeoxycholic acid) into secondary bile acids (deoxycholic acid [DCA] and lithocholic acid) via the 7α‐dehydroxylation pathway. Secondary bile acids are potent signaling molecules that activate the nuclear receptor farnesoid X receptor (FXR) and the G‐protein‐coupled receptor (GPCR) TGR5, thereby influencing host lipid metabolism, glucose homeostasis, and immune responses (Ridlon et al. 2016; Wahlström et al. 2016).

Caveat: Despite the long list of proposed functions, most of these mechanistic assignments derive from in vitro experiments or murine models. Direct evidence in humans for many proposed mechanisms, such as glutathione‐mediated anti‐inflammatory effects or strain‐specific bile acid modifications, remains limited and largely correlational. Furthermore, human studies often rely on fecal metagenomics, which provides taxonomic abundance but does not measure functional activity in situ. Therefore, although the ecological importance of Lachnospiraceae is well established, the translation of these findings to human health requires cautious interpretation.

### 1.3 Current Research Progress and Existing Gaps—A Critical Synopsis

Over the past decade, research on Lachnospiraceae has expanded rapidly, driven by advances in next‐generation sequencing, metabolomics, and gnotobiotic animal models. Several key observations have been consistently reported, but it is crucial to recognize that most of these remain associations rather than proven causal relationships.

**Observed associations (not necessarily causal)**:

1. SCFA‐mediated immune modulation: In rodent models, SCFAs (particularly butyrate) inhibit histone deacetylases (HDACs) and activate GPCR (GPR41, GPR43, and GPR109A), leading to enhanced differentiation of regulatory T cells (Tregs) and follicular regulatory T cells (TFRs) (McBride et al. 2023; Saadh et al. 2025). These effects suppress Th17 responses and have been shown to ameliorate colitis and arthritis in mice.
2. Dietary fiber interventions: Several human randomized controlled trials have demonstrated that supplementation with inulin, resistant starch, or whole grains can increase fecal abundance of Roseburia and Eubacterium and elevate fecal SCFA concentrations (Vandeputte et al. 2017; Vanegas et al. 2017). However, the magnitude of change varies widely among individuals, and fecal SCFA poorly reflects colonic tissue levels.
3. Cross‐sectional human studies: Patients with Type 2 diabetes (T2D), inflammatory bowel disease (IBD), and certain cancers (e.g., melanoma responding to immunotherapy) show altered Lachnospiraceae abundance compared to healthy controls (Haberman et al. 2014; Lu et al. 2022; Lee et al. 2022; Robinson et al. 2023; Macandog et al. 2024; Richie et al. 2024; Gao et al. 2026). These differences are often correlated with immune parameters such as Th1/Th2 ratios, Treg/Th17 balance, or CD8^+^ T cell infiltration.

**Critical gaps that remain unresolved (**Figure 2):

1. Strain‐level functional heterogeneity: The vast majority of studies report data only at the family or genus level, pooling together functionally diverse members. As a result, we do not know which specific strains are beneficial, neutral, or potentially harmful.
2. Causal inference in humans: Almost all human data are cross‐sectional or from uncontrolled intervention studies. With the sole exception of *Anaerobutyricum soehngenii*, for which several RCTs have demonstrated glycemic benefits in metabolic disease populations, there is a striking lack of randomized, double‐blind, placebo‐controlled trials (RCTs) using defined Lachnospiraceae strains with clinical endpoints. The RCT evidence for *A. soehngenii* is strain‐specific and does not extend to other family members or other disease indications.
3. Conflicting evidence: Although many studies report positive associations (e.g., higher Lachnospiraceae linked to better health), some studies have found increased Lachnospiraceae in inflammatory states or after certain environmental exposures (see Section 2.3.2). These contradictions highlight the context‐dependent nature of host‐microbe interactions.
4. Methodological variability: Differences in DNA extraction kits, 16S rRNA primer sets, sequencing platforms (Illumina vs. Nanopore), bioinformatics pipelines (QIIME2, DADA2, and MetaPhlAn), and reference databases (Greengenes, SILVA, and GTDB) hamper cross‐study comparability and meta‐analyses.
5. Lack of functional validation: Most studies infer function from metagenomic gene content (e.g., the presence of butyrate synthesis genes) rather than directly measuring metabolic output (e.g., butyrate production rate using stable isotopes).

**FIGURE 2:**
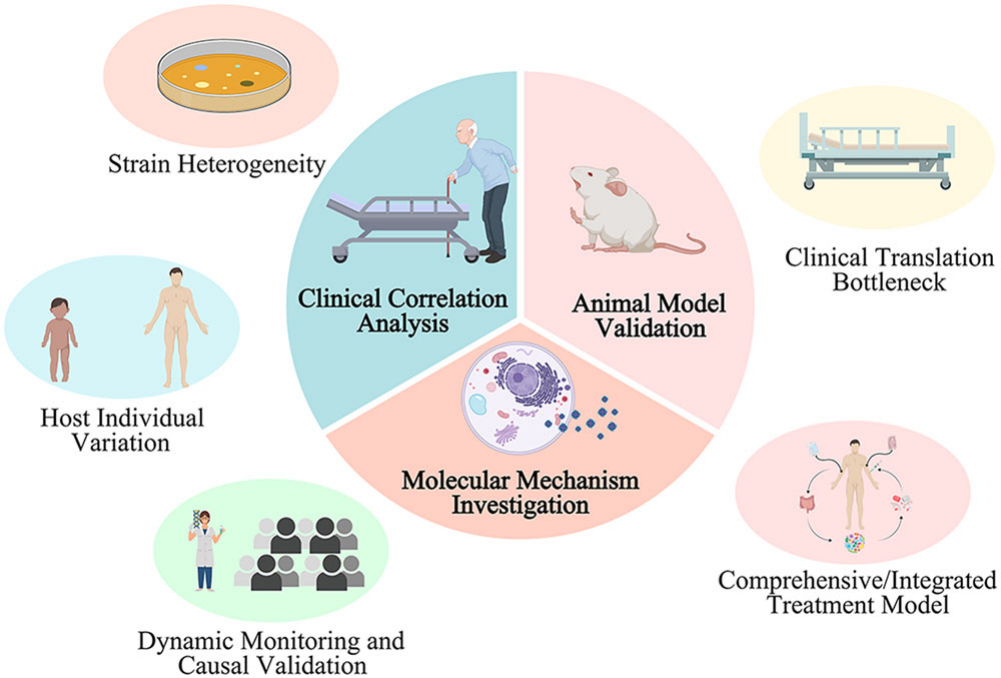
Key research themes and translational barriers in Lachnospiraceae research, covering strain heterogeneity, clinical correlation, animal validation, molecular mechanisms, host variation, causal monitoring, translational bottlenecks, and integrated treatment strategies.

These gaps are elaborated in the following sections, and specific recommendations for future research are provided in Section 6.

## 2 Biological Characteristics of Lachnospiraceae

### 2.1 Classification and Distribution

#### 2.1.1 Taxonomic Classification and Functional Divergence Across Genera and Species

Lachnospiraceae are among the most prevalent anaerobic bacterial groups in the human gut, with relative abundance typically ranging from 15% to 25% in healthy adults, positively correlating with long‐term dietary fiber intake (Meehan and Beiko 2014). Phylogenetic analyses based on 16S rRNA gene sequencing and whole‐genome comparisons have revealed that the family encompasses at least 11 major genera and over 27 distinct species‐level phylogroups, with some estimates exceeding 387 species‐level clusters when including uncultured lineages (Sorbara et al. 2020). Functional differentiation across genera is substantial—SCFA profiles, mucin degradation capacity, and substrate flexibility vary widely. For instance, Roseburia species are highly efficient butyrate producers due to the presence of multiple copies of butyryl‐CoA:acetate CoA‐transferase genes and GH3/GH43 carbohydrate‐active enzymes (CAZymes). In contrast, Blautia species preferentially convert resistant starch and oligosaccharides into propionate via the methylmalonyl‐CoA or acrylate pathways. Coprococcus strains are equipped with mucin‐degrading enzymes (GH20 and GH2), allowing them to associate with the mucus layer, whereas E. rectale additionally synthesizes glutathione for reactive oxygen species (ROS) scavenging.

Crucially, findings from one genus cannot be generalized to the whole family. For example, the strong butyrogenic and anti‐inflammatory effects of R. intestinalis do not imply that Blautia producta (which has been associated with bacteremia in immunocompromised patients) is safe (Mao et al. 2023). Table 2 highlights the striking heterogeneity in evidence quality across Lachnospiraceae genera. Only *A. soehngenii* has RCT data (moderate); *Roseburia* and *E. rectale* rely on correlational evidence (moderate); *Blautia* is very low due to bacteremia case reports; three genera have only limited human data (low). Strain‐specific, not genus‐level, validation is essential.

**TABLE 2:** Overview of functional characteristics and evidence status for major Lachnospiraceae genera.

Genus	Representative strain(s)	Primary SCFA(s)	Key metabolic traits	Evidence level for human translation/benefit	Refs.
*Agathobacter*	*A. rectalis* (formerly *Eubacterium rectale*)	Butyrate + propionate	Flexible metabolism; butyrate and propionate production	Low (limited human data)	Hayashi et al. (2024)
*Anaerobutyricum*	*A. soehngenii* CH106 (formerly *Eubacterium hallii*)	Butyrate	1,2‐propanediol utilization; cross‐feeding; butyrate via butyryl‐CoA:acetate CoA‐transferase	Moderate (GRAS, RCTs for glycemic control)	Shetty et al. (2018), Ilias et al. (2025), FDA GRN 1065
*Anaerobutyricum*	*A. hallii*	Butyrate	Same as above	Low (few human studies)	Shetty et al. (2018)
*Blautia*	*B. producta*, *B. obeum*	Acetate (and minor propionate)	Hydrogen utilization; acetate production; methylmalonyl‐CoA/acrylate pathway	Very low (case reports of bacteremia; no efficacy trials)	Liu et al. (2021), Bodin et al. (2024), Zhao et al. (2025).
*Coprococcus*	*C. catus*, *C. eutactus*	Mixed (butyrate + propionate)	Mucin‐degrading enzymes (GH20 and GH2)	Low (limited human data)	Tailford et al. (2015), Bell and Juge (2021)
*Dorea*	*D. formicigenerans*	Mixed	Diverse CAZymes for polysaccharide degradation	Low (few human studies)	Dahl et al. (2020)
*Eubacterium*	*E. rectale*	Butyrate	Butyrate production; resistant starch fermentation; also synthesizes glutathione	Moderate (animal + human correlational)	Vacca et al. (2020), Richie et al. (2024)
*Roseburia*	*R. intestinalis*, *R. hominis*	Butyrate	Butyryl‐CoA:acetate CoA‐transferase; dietary fiber degradation	Moderate (animal + human correlational)	Louis and Flint (2017), Duncan et al. (2002)

Abbreviations: FDA, Food and Drug Administration; GRAS, generally recognized as safe; RCT, randomized controlled trial; SCFA, short‐chain fatty acid.

#### 2.1.2 Distribution Patterns in Health and Disease—Association vs. Causation

Numerous cross‐sectional studies have reported altered Lachnospiraceae abundance in various disease states. It is essential to emphasize that these are associations, not proven causal relationships. Confounding factors—including diet, medications (especially antibiotics, proton pump inhibitors, and metformin), disease stage, comorbidities, and differences in sequencing methods—are rarely adequately controlled.

- T2D: Compared with healthy controls, fecal samples from T2D patients exhibit significantly reduced abundances of SCFA‐producing taxa, including the Lachnospiraceae NK4A136 group, Ruminococcaceae UCG‐002, and Eubacterium hallii group. Notably, peripheral Th1/Th2 and Th17 cell ratios are positively correlated with the abundances of these bacterial groups, suggesting a potential mediating role in diabetes‐associated chronic inflammation. However, reverse causality is possible: Hyperglycemia itself may alter gut microbiota composition.
- IBD: Pediatric Crohn's disease (CD) patients display characteristic dysbiosis early in disease progression, marked by increased Enterobacteriaceae and concurrent decreases in Lachnospiraceae and Ruminococcaceae. Multivariate analyses confirm a significant negative correlation between Lachnospiraceae abundance and IFN‐γ levels, implicating the reduction of Lachnospiraceae in promoting Th1‐mediated mucosal inflammation (Haberman et al. 2014). Longitudinal studies suggest that this dysbiosis precedes clinical flares in some patients, but not all, indicating inter‐individual variability.
- Obesity and metabolic syndrome: Nlrp12‐deficient mice fed a high‐fat diet exhibit increased body mass index, impaired insulin tolerance, reduced intestinal Lachnospiraceae abundance, and decreased expression of SCFA synthesis enzymes. These alterations correlate with heightened low‐grade inflammation in liver and adipose tissue (Lau and Dombrowski 2018). Important caveat: This is an animal model study—human data are limited to cross‐sectional correlations. A recent meta‐analysis of 17 human cohorts found no consistent direction of change for Lachnospiraceae in obesity, suggesting that family‐level analyses are too coarse to capture meaningful associations (Duvallet et al. 2017; Chanda and De 2024; Vishwakarma et al. 2025).
- Cancer: In immunocompetent mice, intratissue Ruminococcus gnavus and B. producta (both Lachnospiraceae) suppressed colorectal carcinoma growth and promoted CD8^+^ T cell activation. Mechanistically, these strains degraded lyso‐glycerophospholipids that inhibit CD8^+^ T cell activity, thereby preserving immune surveillance (Zhang et al. 2023). No human data are currently available to confirm these findings in patients. Moreover, B. producta has been isolated from blood cultures in immunocompromised patients, raising hypothetical safety concerns that warrant investigation (Bodin et al. 2024). However, these events are rare and have not been systematically quantified.

Critical note: Causal relationships have not been established in humans. Longitudinal prospective studies (pre‐disease baseline sampling) and intervention trials (e.g., fiber supplementation followed by metagenomic and immunological monitoring) are urgently needed to determine whether altered Lachnospiraceae abundance is a cause, a consequence, or an innocent bystander of disease.

### 2.2 Metabolic Functions and SCFA Production—From Dietary Substrates to Functional Outcomes

#### 2.2.1 Dietary Fiber Substrates and Selective Fermentation

Lachnospiraceae are specialized fermenters of dietary fibers, but not all fibers are equal. The chemical structure (linear vs. branched, degree of polymerization), solubility, and physical encapsulation of a fiber within the food matrix determine which Lachnospiraceae taxa can degrade it and what SCFA profile is produced. Table 3 summarizes the most relevant substrate–microbe–SCFA relationships for functional food design, along with the level of evidence supporting each association.

**TABLE 3:** Dietary substrates, primary responders, and SCFA profiles—Evidence levels.

Fiber type	Primary responders	Dominant SCFA	Evidence strength	Refs.
Inulin/FOS	Roseburia, Blautia	Acetate, butyrate	Moderate (human trials)	Vandeputte et al. (2017)
Pectin	Lachnospira pectinoschiza, some Roseburia	Acetate → butyrate (delayed)	Low (animal only)	Wu et al. (2022)
Resistant starch (RS)	E. rectale, R. intestinalis	High butyrate	Moderate (in vitro + animal)	Louis and Flint (2017), Klostermann et al. (2023)
Arabinoxylan	Roseburia, Blautia	Acetate, butyrate	Low‐moderate (human correl.)	Vanegas et al. (2017)
Fiber mixtures	Multiple genera	Synergistic SCFA increase	Emerging (needs replication)	Opperman et al. (2026)

Abbreviations: FOS, fructo‐oligosaccharides; SCFA, short‐chain fatty acid.

Inulin and fructo‐oligosaccharides (FOS): These soluble, prebiotic fibers are rapidly fermented by Roseburia and Blautia species, yielding high acetate and butyrate levels (Zhang et al. 2024). In a human randomized crossover trial, inulin supplementation (16 g/day for 2 weeks) significantly increased fecal Roseburia abundance and butyrate concentration, but the effect was highly individual, with only 60% of participants showing a response (Vandeputte et al. 2017).

Pectin: This complex polysaccharide is degraded by Lachnospira pectinoschiza and certain Roseburia strains. Pectin fermentation produces acetate first, followed by delayed butyrate production via cross‐feeding with other bacteria (e.g., Eubacterium species). In a Clostridioides difficile mouse model, replacing insoluble fiber (cellulose) with pectin increased Lachnospiraceae abundance, elevated cecal butyrate and propionate, reduced inflammatory markers, and improved epithelial barrier function (Kalita et al. 2025). Human data are limited to in vitro fecal fermentations; no human intervention trial has specifically examined pectin‐Lachnospiraceae interactions in vivo.

Resistant starch Type 3 (RS type 3): RS3 is a potent butyrogenic substrate. It is primarily fermented by E. rectale, R. intestinalis, and Anaerostipes species. The butyrate yield from RS3 is among the highest of all fibers, as demonstrated in in vitro fecal fermentation systems (Louis and Flint 2017). A small human trial (*n* = 20) reported that RS3 supplementation (30 g/day for 3 weeks) increased fecal E. rectale abundance and butyrate, but no clinical outcomes were measured (Klostermann et al. 2023). Few human RCTs have tested RS interventions with strain‐level monitoring.

Arabinoxylan: Found in whole grains (wheat, rye, and barley), this fiber is selectively utilized by Roseburia and Blautia species, producing acetate and butyrate. A 6‐week randomized controlled trial substituting whole‐grain wheat for refined wheat significantly increased fecal Roseburia abundance and butyrate, but changes in immune markers (IL‐6, C‐reactive protein [CRP], tumor necrosis factor alpha [TNF]‐α) were modest and not statistically significant after multiple testing correction (Vanegas et al. 2017).

Fiber mixtures: Importantly, fiber blends often produce synergistic SCFA outputs exceeding the sum of individual components. This is due to cross‐feeding among primary degraders (e.g., Bifidobacterium breaking down inulin into fructose oligomers) and secondary butyrate producers (Lachnospiraceae converting lactate/acetate to butyrate). For example, a mixture of inulin, RS3, and pectin produced significantly higher butyrate than any single fiber in an in vitro gut model (Opperman et al. 2026). However, human validation is lacking, and the optimal ratios likely vary between individuals depending on baseline microbiota composition.

Caveat: Most human studies use fecal metagenomics and fecal SCFA measurements, which poorly reflect colonic tissue concentrations or in situ metabolic activity. SCFAs are rapidly absorbed, and fecal levels represent only the excess that escapes absorption. Stable isotope tracer studies (e.g., using ^13^C‐labeled fibers) are needed to quantify true production rates.

#### 2.2.2 SCFA Biosynthetic Pathways—Concise Overview (Food‐Relevant)

For readers interested in the biochemical details, we provide a brief summary of the two main SCFA synthesis pathways that are most relevant to food fermentation and probiotic development:

- Butyrate is produced predominantly via the butyryl‐CoA:acetate CoA‐transferase pathway. Acetyl‐CoA derived from glycolysis is condensed to acetoacetyl‐CoA, reduced to butyryl‐CoA, and then the CoA moiety is transferred to acetate, yielding butyrate. This pathway is present in Roseburia, E. rectale, Faecalibacterium prausnitzii, and Anaerostipes species. An alternative butyrate kinase (buk) pathway is less common in Lachnospiraceae.
- Propionate is synthesized via the succinate pathway (from succinate to propionate, involving methylmalonyl‐CoA) or the acrylate pathway (direct reduction of lactate to propionate). The acrylate pathway is prominent in Blautia species and allows them to produce propionate from lactate, which is a key cross‐feeding interaction.

Fermentation efficiency is influenced by luminal pH, substrate concentration, and transit time. Notably, when pH drops below 6.0 (e.g., due to acetate accumulation), butyrate‐producing bacteria are favored, creating a positive feedback loop that further enhances butyrate production (Louis and Flint 2017). This has practical implications for functional food design: adding buffering agents or combining fibers with different fermentation kinetics may help maintain a favorable pH.

#### 2.2.3 Implications for Food Processing and Functional Food Development

Food processing methods (extrusion, cooking, grinding, and milling) alter fiber structure and accessibility in ways that can profoundly affect SCFA production. For example, extrusion (high temperature and high shear) can disrupt the physical encapsulation of fibers within the plant cell wall, making them more rapidly fermentable. However, very rapid fermentation may lead to SCFA production in the proximal colon rather than the distal colon, where butyrate is most needed for barrier function and immune regulation (Smith et al. 2022). Fine milling of whole grains increases the surface area for enzymatic attack, increasing fermentability but also potentially accelerating glucose release, which could be undesirable for glycemic control. Particle size reduction generally increases fermentation rate but may reduce butyrate yield if substrates are too rapidly degraded and consumed by other bacteria before Lachnospiraceae can utilize them.

Rational design of functional foods should consider fiber mixtures that exploit cross‐feeding to maximize butyrate output. For example, a combination of a rapidly fermented fiber (e.g., inulin) and a slowly fermented fiber (e.g., RS3) may provide a sustained supply of fermentable substrate to distal gut sections. However, most formulation studies are in vitro; human validation is sparse. A note has been added to Table 4: “Most data from in vitro fermentation; human confirmation needed.” Furthermore, food matrices (e.g., whole grains vs. isolated fibers) alter fermentation outcomes, and more research is needed on how different food formulations affect Lachnospiraceae metabolism.

**TABLE 4:** Evidence grading for additional immune mechanisms.

Mechanism	Direct evidence in Lachnospiraceae?	Direct evidence in other taxa?	Proposed role for Lachnospiraceae	Refs.
Flagellin → TLR5 → Treg	Yes (a few strains)	Yes (e.g., Escherichia coli)	Likely strain‐specific, not generalizable	Crellin et al. (2005), Flores‐Langarica et al. (2018)
EPS → immune modulation	No	Yes (Bacteroides fragilis)	Hypothetical, requires validation	Shen et al. (2012), Round and Mazmanian (2010)
7α‐dehydroxylation → LCA/DCA	Yes (established)	Yes	Established core function	Ridlon et al. (2016)
LCA/DCA → FXR/TGR5 → anti‐inflammatory	No (host response)	Yes (cell lines, mice)	Mediated by host, independent of bacteria	Fiorucci et al. (2018), Pols et al. (2011)
3‐oxoLCA/isoalloLCA → Th17/Treg	No	Yes (Bacteroides, Clostridium)	Unlikely direct; possible cross‐feeding	Hang et al. (2019), Paik et al. (2022)

Abbreviations: DCA, deoxycholic acid; EPS, extracellular polysaccharides; FXR, farnesoid X receptor.

### 2.3 Taxonomic Framework and the Imperative of Strain‐Level Attribution

A fundamental premise of this review is that the Lachnospiraceae family is phylogenetically and functionally heterogeneous (Pardesi et al. 2023; Rainey et al. 2015). The family, established in 2009 with *Lachnospira* as the type genus, belongs to the Clostridial cluster XIVa of the phylum Bacillota and comprises over 82 valid genera and 176 valid species at the time of writing (Pardesi et al. 2023). Ongoing taxonomic reclassifications—such as the transfer of *E. hallii* to *A. soehngenii* (Shetty et al. 2018) and the reclassification of certain *Ruminococcus* species to *Mediterraneibacter* (Schaus et al. 2024)—underscore the dynamic nature of the taxonomy within this family.

To ensure consistency throughout this review, we adopt the GTDB as our primary taxonomic framework (Parks et al. 2022), while cross‐referencing with the List of Prokaryotic names with Standing in Nomenclature (LPSN) where historical nomenclature is used in cited studies. The GTDB framework provides a phylogenomic approach to taxonomy that is particularly valuable for resolving the polyphyletic nature of genera such as *Ruminococcus*, which contains species belonging to both the Lachnospiraceae and Ruminococcaceae families (Schaus et al. 2024; Molinero et al. 2022).

A critical caveat must be stated at the outset: Metabolic and immunomodulatory functions are not family‐ or even genus‐wide properties. The available evidence demonstrates that

- Propionate production, for example, is a characteristic of specific species such as *Anaerotignum propionicum* and the recently described *Chakrabartyella piscis* (Pardesi et al. 2023), not a general feature of Lachnospiraceae.
- Glutathione synthesis has been demonstrated specifically in E. rectale, which utilizes a noncanonical pathway involving genes homologous to yliA/busA rather than classical gshA/gshB (Richie et al. 2024, 2026). This function is not shared by other Lachnospiraceae strains tested (Richie et al. 2026).
- Mucin degradation is a specialized function of certain species such as *Ruminococcus torques* (recently reclassified as *Mediterraneibacter torques*) (Schaus et al. 2024), which acts as a keystone degrader of intestinal mucin glycoprotein (Schaus et al. 2024). Other Lachnospiraceae members, such as Lachnospiraceae bacterium oral taxon 082, possess putative mucin‐desulfating sulfatases, but this is not a family‐wide trait.
- Bile acid metabolism—including 3α‐hydroxysteroid dehydrogenase (HSDH), 3β‐HSDH, and 5β‐reductase activities—has been demonstrated in specific unclassified Lachnospiraceae strains (St57, St58, and St62) isolated from centenarian microbiomes (Yuko et al. 2021) but cannot be generalized to the entire family.

Throughout the remainder of this review, we therefore explicitly attribute functional claims to the specific species or strain for which evidence exists, and we avoid generalizations about “Lachnospiraceae” as a functional unit. Where the literature itself has made such generalizations, we critically evaluate this as a limitation and emphasize the need for strain‐level resolution.

### 2.4 Symbiosis and Pathogenicity—A Context‐Dependent Continuum

#### 2.4.1 Beneficial Functions Under Homeostatic Conditions

Under normal physiological conditions, SCFA‐producing Lachnospiraceae contribute to barrier integrity and immune homeostasis through multiple integrated mechanisms: (i) upregulation of tight junction proteins (ZO‐1, occludin, and claudin‐1) and MUC2 mucin secretion, which physically strengthens the epithelial barrier; (ii) HDAC inhibition and GPCR activation leading to increased production of anti‐inflammatory cytokines (IL‐10 and transforming growth factor beta [TGF‐β]) and suppression of pro‐inflammatory mediators; and (iii) competitive exclusion of opportunistic pathogens (e.g., C. difficile and Salmonella) via niche occupation, SCFA‐mediated pH reduction, and secretion of bacteriocins (Louis and Flint 2017; Vacca et al. 2020).

#### 2.4.2 Context‐Dependent Pathogenic Potential and Safety Risks

Contradictory evidence exists: Although the vast majority of studies highlight beneficial roles, several reports indicate that under specific conditions, some Lachnospiraceae members can shift toward a pathogenic or pro‐inflammatory phenotype. This context dependency is critical for safety assessment (Figure 3).

1. Environmental triggers: In mice exposed to the industrial solvent trichloroethylene and microplastics, increased Lachnospiraceae abundance paradoxically correlated with elevated colonic ROS and pro‐inflammatory markers (CD14 and IL‐1β) and decreased tight junction proteins, contributing to systemic autoimmunity as well as the decrease in butyrate production and a decrease in SCFA levels (Wang et al. 2021; Yang et al. 2026) (Figure 3A). This suggests that the same bacterial family can have opposite effects depending on host exposure.
2. Antibiotic resistance genes: Genomic surveys have identified acquired antibiotic resistance determinants—including tetracycline (tetM and tetW), macrolide (ermB), and aminoglycoside resistance genes—in some Lachnospiraceae isolates (Juricova et al. 2021).
3. Clinical case reports: *B. producta* has been isolated from blood cultures in immunocompromised patients (e.g., acute myeloid leukemia and posttransplant) (Bodin et al. 2024). However, the absolute incidence of such events is unknown; population‐level epidemiological data are not available, and the estimated frequency should not be interpreted as a precise risk estimate and occur almost exclusively in severely immunocompromised hosts.

**FIGURE 3:**
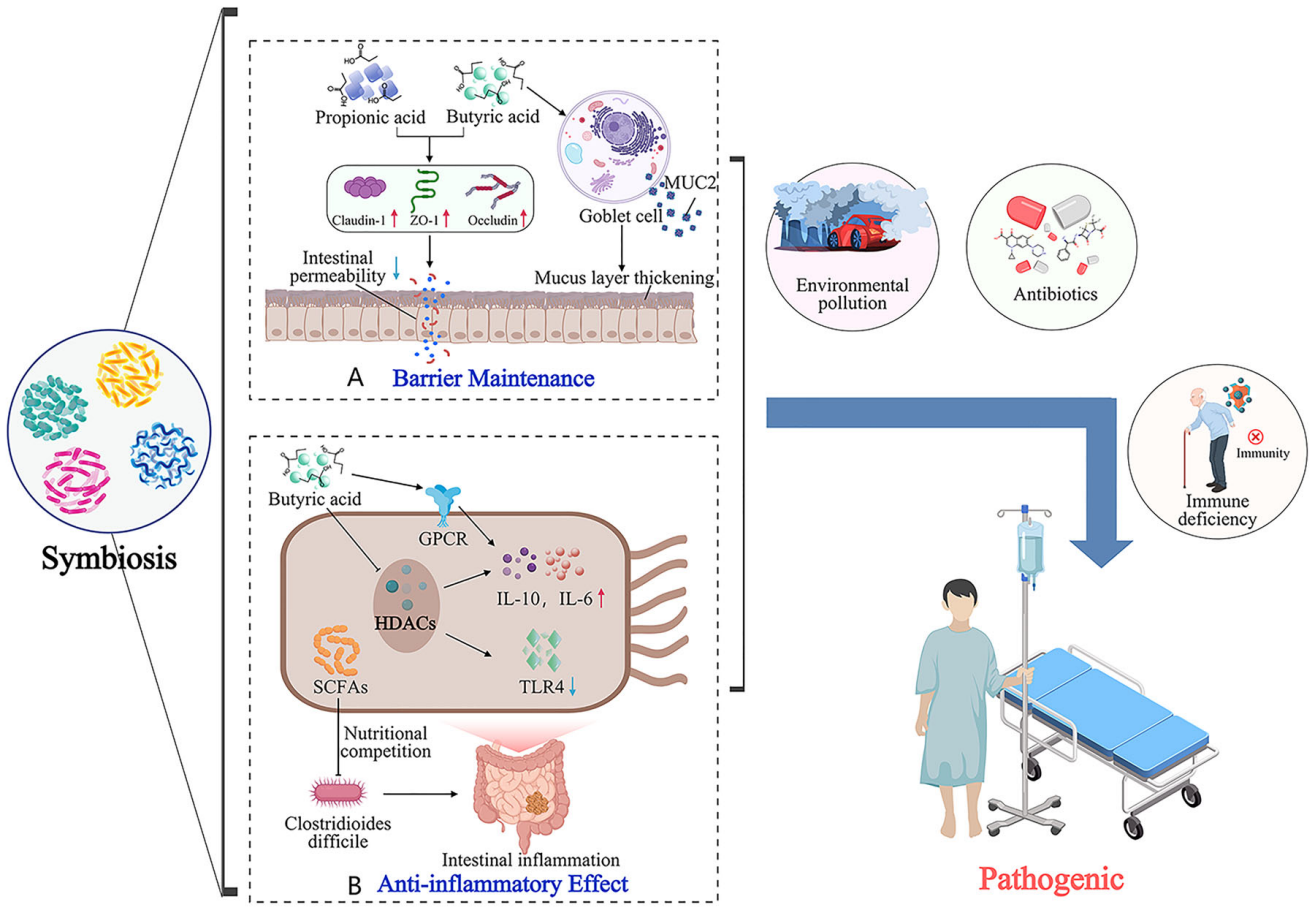
Overview of symbiotic and pathogenic roles of butyrate‐producing bacteria in intestinal health. (A) Depicts barrier maintenance via SCFA‐mediated upregulation of tight junctions (ZO‐1, occludin), mucus thickening, GPCR/HDAC signaling, and competitive exclusion of *Clostridioides difficile*. (B) Summarizes anti‐inflammatory effects of SCFAs and factors (pollution, antibiotics, immune deficiency) that disrupt symbiosis. It illustrates the pathogenic potential when host defenses are compromised, leading to barrier breakdown and systemic inflammation. This framework emphasizes the context‐dependent nature of host–microbe interactions in the Lachnospiraceae family. GPCR, G‐protein‐coupled receptor; HDAC, histone deacetylase; IL, interleukin; TLR4, toll‐like receptor 4; ZO‐1, zonula occludens‐1.

Important: These pathogenic associations appear to be rare and context‐dependent based on available case reports, but systematic epidemiological data are lacking. They do not negate the general beneficial role of most Lachnospiraceae members in healthy individuals (Ravikrishnan et al. 2024). However, they underscore the need for strain‐level safety assessment before any Lachnospiraceae‐based product is used in foods or therapies.

#### 2.4.3 Strain‐Specific Functional Heterogeneity—A Major Unresolved Issue

Not all Lachnospiraceae are equal. Substantial functional diversity exists between genera and even among strains of the same species. On the basis of current evidence, we propose a three‐tier classification:

1. High‐confidence beneficial strains: R. intestinalis and E. rectale consistently show strong butyrate production, HDAC inhibition, and anti‐inflammatory effects in vitro and in vivo, with no reported safety issues in healthy animals or humans (Sorbara et al. 2020; Vacca et al. 2020) (Figure 3B). These are the most promising candidates for further development.
2. Mixed or insufficient evidence: Several Blautia species (e.g., Blautia obeum and Blautia wexlerae) are associated with healthy states and metabolic benefits in some studies, whereas B. producta has been linked to bacteremia and antibiotic‐associated diarrhea (Bodin et al. 2024). Similarly, Dorea abundance fluctuates in IBD and metabolic syndrome, with context‐dependent roles that are not yet understood (Maharshak et al. 2018).
3. Low risk but under‐characterized: Coprococcus, Anaerostipes, and Butyrivibrio are generally considered beneficial based on their SCFA profiles, but strain‐level safety data are extremely limited. No human intervention studies have been performed with pure cultures of these genera.

Collectively, Strain‐level characterization—including whole‐genome sequencing (to screen for antibiotic resistance and virulence genes), in vitro safety assays (hemolysis, gelatinase activity, and biogenic amine production), and functional immune assays—is mandatory before any Lachnospiraceae‐based product can be considered for food or therapeutic use.

#### 2.4.4 Regulatory Considerations for Next‐Generation Probiotics

To date, only A. soehngenii CH106 has received generally recognized as safe (GRAS) status in the United States (US) (Food and Drug Administration [FDA] GRN No. 1065, April 2023), and this designation applies strictly to its use as a food ingredient, not to therapeutic claims. No other Lachnospiraceae strain possesses GRAS status, and none has qualified presumption of safety (QPS) status in the EU. The GRAS status of A. soehngenii CH106 does not imply that other strains are safe or that regulatory approval is easily obtained for other members of the family. This is a major barrier for food applications. The absence of established safety recognition means that any company wishing to market a Lachnospiraceae‐containing food product would need to submit a novel food application or a GRAS notification, requiring extensive safety data. The required steps for approval typically include:

- Whole‐genome sequencing to screen for acquired antibiotic resistance genes and virulence factors.
- In vitro safety assays (hemolysis, gelatinase, d‐lactate, biogenic amine production).
- In vivo toxicity studies (e.g., 28‐day oral toxicity study in rodents).
- If intended for therapeutic use as a live biotherapeutic product (LBP), an investigational new drug (IND) application and Phases I–III clinical trials are required (Spacova et al. 2023).

Given these hurdles, pragmatic near‐term alternatives include postbiotics—preparations of heat‐killed cells (or their structural components) that confer health benefits—and metabolite‐based preparations (e.g., purified SCFAs or fermentation‐derived metabolites). Importantly, under the International Scientific Association for Probiotics and Prebiotics (ISAPP) definition, postbiotics must contain inactivated microbial cells or their components; purified metabolites without cellular biomass do not qualify as postbiotics and are referred to here as metabolite‐based preparations (Salminen et al. 2021). Postbiotics are not subject to the same stringent regulatory requirements and can be marketed as food ingredients with a simpler safety dossier.

## 3 Mechanisms of Immune Regulation—A Unified Critical Framework

Note on organization: To eliminate redundancy and improve clarity, this section presents a unified framework for all immunomodulatory mechanisms attributed to Lachnospiraceae and their metabolites (Figure 4A). Detailed descriptions of SCFA biochemistry (HDAC inhibition and GPCR activation) are provided once in Section 3.1. Subsequent Sections (3.2–3.4) refer back to these mechanisms rather than reiterating them. Lengthy enumerations of individual animal studies have been consolidated into summary statements with explicit evidence grading. The mechanisms described here provide the foundational framework for understanding how Lachnospiraceae may influence disease pathogenesis; however, as detailed in Section 5, the translation of these preclinical mechanisms to human disease contexts remains largely unproven.

**FIGURE 4:**
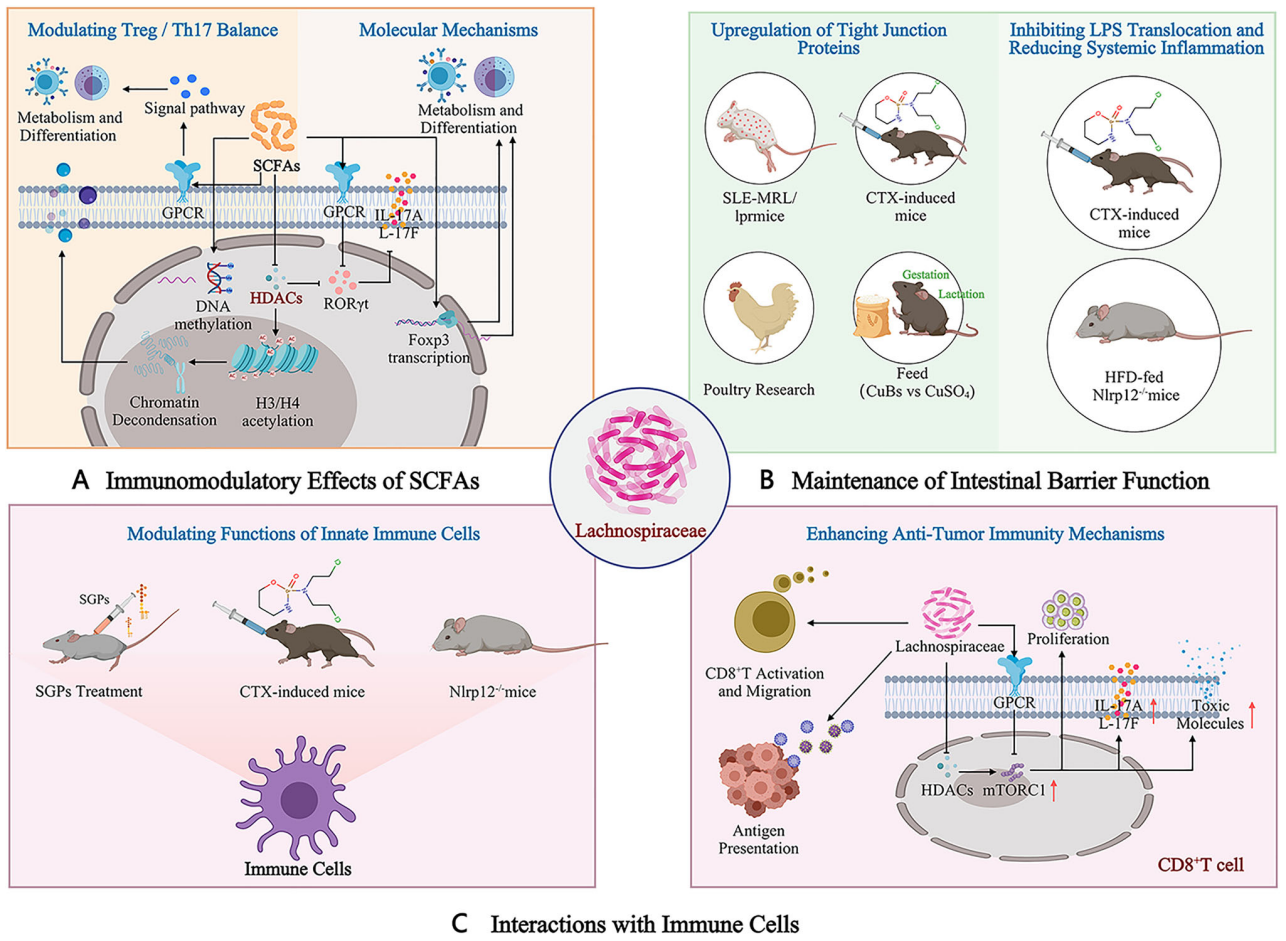
Schematic illustration of the functional mechanisms of Lachnospiraceae in immunomodulation, intestinal barrier maintenance, and immune cell interactions. (A) Immunomodulatory effects of SCFAs: SCFAs produced by Lachnospiraceae participate in the regulation of epigenetic modifications (such as DNA methylation, H3/H4 acetylation, and chromatin decondensation) by binding to GPCRs and inhibiting HDACs. These molecular mechanisms further regulate downstream signaling pathways, influencing cellular metabolism and differentiation. Ultimately, they modulate the balance between Treg and Th17 cells and control the expression of key transcription factors (e.g., RORγt and Foxp3). (B) Maintenance of intestinal barrier function: Lachnospiraceae contributes to the upregulation of tight junction proteins and mitigates systemic inflammation by inhibiting lipopolysaccharide (LPS) translocation. These protective effects have been demonstrated across various animal models, including SLE‐MRL/lpr mice (He et al. 2024), CTX‐induced mice, poultry models, gestating/lactating mice, and HFD‐fed Nlrp12^−/−^ mice. (C) Interactions with immune cells: Modulation of innate immune cell functions: In specific treatment contexts (e.g., SGPs and CTX‐induction) or Nlrp12^−/−^ mouse models, Lachnospiraceae regulates the activity of innate immune cells. Enhancement of antitumor immunity mechanisms: Lachnospiraceae promotes the activation, migration, and proliferation of CD8+ T cells through GPCR signaling pathways and HDACs/mTORC1‐mediated mechanisms. Concurrently, it upregulates the expression of cytotoxic molecules (such as *IL‐2*, *IFN‐γ*, and *TNF‐α*) and effector toxins, while enhancing antigen presentation, collectively contributing to antitumor immune responses. GPCR, G‐protein‐coupled receptor; HDAC, histone deacetylase; IL, interleukin; SCFAs, short‐chain fatty acids; TLR4, toll‐like receptor 4.

### 3.1 Immunomodulatory Effects of SCFAs—Critical Appraisal of the Evidence

#### 3.1.1 Molecular Mechanisms: HDAC Inhibition and GPCR Activation—Evidence and Caveats

SCFAs (acetate, propionate, and butyrate) have been shown to inhibit histone deacetylases (HDACs) and to activate GPCR (GPR41, GPR43, and GPR109A) in in vitro and murine studies. These effects enhance acetylation of histones H3 and H4 at the Foxp3 gene promoter, leading to chromatin relaxation and increased transcription of anti‐inflammatory genes (*IL‐10* and *IL‐22*). Consequently, they promote the differentiation of regulatory T cells (Tregs) and follicular regulatory T cells (TFRs) (Furusawa et al. 2013; Campbell et al. 2013). In a collagen‐induced arthritis (CIA) mouse model, microbial‐derived butyrate significantly enhanced histone acetylation in the promoter regions of TFR cell lineage‐defining genes (e.g., Bcl‐6 and Foxp3), increased functional TFR cell numbers, reduced pathological autoantibody production, and alleviated synovial inflammation and bone destruction (Takahashi et al. 2020).

However, several important caveats apply before extrapolating these findings to humans. Highlights four critical caveats that significantly impede the direct translation of SCFA research from preclinical models to human clinical applications. First, dose relevance is a primary concern; most in vitro assays employ supraphysiological butyrate concentrations (0.5–5 mM) that far exceed the estimated physiological levels in the lamina propria (0.1–1 mM) (Jiang, Incarnato, et al. 2025), where immune cells reside, and the majority of luminal SCFAs are metabolized by colonocytes before reaching systemic circulation. Second, substantial species differences exist in the expression and signaling of SCFA receptors; robust functional outcomes such as Treg induction, which are consistently observed in murine cells, are considerably weaker and more variable in human peripheral blood mononuclear cells (PBMCs). Third, the literature presents conflicting findings, particularly regarding propionate, which at high concentrations can promote pro‐inflammatory Th17 differentiation, illustrating that SCFA signaling is highly context‐dependent on the local cytokine milieu (e.g., IL‐6 and TGF‐β). Finally, there is a striking scarcity of direct human data; to date, no study has successfully measured histone acetylation in Tregs isolated from colonic biopsies before and after a high‐fiber intervention. Consequently, the causal chain linking dietary SCFA increases to epigenetic modulation in human immune cells remains entirely hypothetical. Collectively, these limitations underscore the urgent need for advanced translational models, rigorous dose‐finding studies, and direct tissue‐level measurements in humans to validate the therapeutic potential of SCFAs.

#### 3.1.2 Other SCFA‐Related Mechanisms—Summary With Evidence Grading

To avoid redundancy, we summarize additional mechanisms with explicit evidence grading.

Flagellin‐toll‐like receptor 5 (TLR5) pathway: Many Lachnospiraceae members are motile and produce flagellin, a canonical ligand for TLR5. In murine models, flagellin from specific Roseburia strains activates CD103^+^ dendritic cells (DCs), which then migrate to mesenteric lymph nodes and secrete TGF‐β and retinoic acid, promoting Treg differentiation (Kinnebrew et al. 2012; Round and Mazmanian 2010). Caveat: This mechanism has been demonstrated for a few strains only; generalization to the whole family is not justified. Moreover, flagellin can also be pro‐inflammatory under certain conditions (e.g., when the epithelial barrier is disrupted).

Extracellular polysaccharides (EPS): EPS can physically shield immunostimulatory surface molecules (e.g., peptidoglycan and lipoteichoic acid) from recognition by host pattern recognition receptors, dampening excessive inflammation (Flemming et al. 2025). In other commensals (e.g., Bacteroides fragilis polysaccharide A), EPS induces Tregs via TLR2 (Kinnebrew et al. 2012). However, no EPS from any Lachnospiraceae member has been functionally characterized for immunomodulatory activity to date. Genome mining reveals EPS biosynthesis clusters, but direct testing is lacking. Therefore, any proposed role for Lachnospiraceae EPS remains hypothetical (Yang, Ren, et al. 2025).

Bile acid metabolites: Lachnospiraceae are well‐established mediators of secondary bile acid production via the 7α‐dehydroxylation pathway, converting primary bile acids (cholic acid and chenodeoxycholic acid) into secondary bile acids (DCA and lithocholic acid) (Wahlström et al. 2016). Secondary bile acids activate the nuclear receptor FXR (in intestinal epithelial cells) and the GPCR TGR5 (on macrophages and DCs), which together suppress NF‐κB signaling and reduce pro‐inflammatory cytokine production (Wahlström et al. 2016). However, the more recently discovered immunomodulatory derivatives—3‐oxolithocholic acid (3‐oxoLCA) and isoallolithocholic acid (isoalloLCA)—which regulate Th17/Treg balance, have been primarily characterized in other bacterial groups (e.g., Bacteroides, Clostridium clusters XIVa and XVIII) (Hang et al. 2019; Tong and Lou 2025). Direct evidence that Lachnospiraceae produce 3‐oxoLCA or isoalloLCA is currently lacking. They may contribute indirectly via cross‐feeding, but this remains speculative.

### 3.2 Maintenance of Intestinal Barrier Function—Integrated Summary

Multiple murine studies have shown that SCFAs (particularly butyrate) upregulate the expression of tight junction proteins (ZO‐1, occludin, and claudin‐1) via activation of AMPK and mitogen‐activated protein kinase (MAPK) signaling pathways and reduce the translocation of lipopolysaccharide (LPS) from the gut lumen into the circulation (Wang et al. 2012; Boets et al. 2016) (Figure 4B). For example, interventions that increase Lachnospiraceae abundance—such as dietary fiber (pectin and inulin), Weizmannia coagulans supernatants, or Cordyceps protein extracts—have been reported to improve barrier integrity in rodent models of lupus, immunosuppression, and high‐fat diet‐induced metabolic syndrome (Truax et al. 2018; Gonzalez et al. 2019; Yang, Fu, et al. 2025).

Critical caveats:

- Most studies use indirect readouts (fecal SCFA, 16S sequencing, or serum LPS) and do not measure barrier function directly in humans (e.g., using the lactulose/mannitol ratio in urine or confocal laser endomicroscopy).
- Causal evidence from human interventions is lacking; no RCT has demonstrated that increasing Lachnospiraceae abundance improves gut barrier function in healthy individuals or patients.
- Poultry and rodent data may not translate directly due to differences in gut physiology, microbiota composition, and diet. For example, the human colon is longer and more compartmentalized than the mouse colon, and the thickness of the mucus layer differs (McBride et al. 2023).

Collectively, the barrier protective effects of SCFAs are well documented in animals, but human translation remains unproven. High‐fiber diets are safe and recommended for general health, but attributing barrier improvements specifically to Lachnospiraceae (rather than to other SCFA producers such as Faecalibacterium) is not yet justified.

### 3.3 Direct Interaction With Immune Cells—Consolidated Evidence

#### 3.3.1 Regulation of Innate Immune Cell Function

In rodent models, SCFAs promote M2 (anti‐inflammatory) macrophage polarization and limit DC maturation, as shown in Nlrp12‐deficient mice and in immunosuppression models (Truax et al. 2018; Li et al. 2024). In the Nlrp12‐deficient mouse model, high‐fat diet feeding combined with microbial dysbiosis synergistically promoted M1‐type pro‐inflammatory macrophage polarization and hyperactivation of NF‐κB/MAPK signaling. Supplementation with Lachnospiraceae or SCFAs reversed M1 polarization, increased the proportion of M2‐type macrophages, suppressed pro‐inflammatory cytokine secretion, and improved metabolic parameters (Truax et al. 2018). SCFAs are also reported to modulate DC function via HDAC inhibition and GPCR activation, interfering with fractalkine receptor (CX3CR1) expression on DCs, which prevents their migration to lymph nodes and reduces IL‐12 secretion, thereby limiting the initiation of excessive adaptive immune responses (Liu et al. 2012).

Critical caveats:

- These mechanisms have been demonstrated almost exclusively in rodents. Human data are limited to small ex vivo studies (e.g., treating monocyte‐derived DCs from healthy volunteers with butyrate), which show variable effects.
- The relevance of these findings to dietary interventions in healthy humans is unknown. Most rodent studies use supraphysiological SCFA concentrations (e.g., 5–10 mM in drinking water) that are not achievable through diet alone.
- The interplay between SCFA‐mediated effects on innate cells and the broader microbiota community is complex and not well captured in reductionist models.

#### 3.3.2 Antitumor Immunity—Correlative Evidence Only

Immune evasion within the tumor microenvironment is a major obstacle limiting the efficacy of cancer immunotherapies. Enhancing tumor‐infiltrating immune cell function through microbiota modulation has emerged as a promising area of research. Multicenter retrospective studies have reported positive correlations between Lachnospiraceae abundance (e.g., the Lachnospiraceae_UCG_010 group) and CD8^+^ T cell infiltration in patients responding to immune checkpoint inhibitors (ICIs) (Zhang et al. 2023; Pu et al. 2024). For example, in a cohort of 158 patients with stage III/IV melanoma, non‐small cell lung cancer (NSCLC), and renal cell carcinoma, the fecal microbiota of ICI responders was enriched in Oscillospira, Clostridia_UCG_014, and Lachnospiraceae_UCG_010, and the abundance of these genera positively correlated with peripheral blood and tumor‐infiltrating CD8^+^ T cell counts, as well as with IFN‐γ and TNF‐α expression (Robinson et al. 2023; Macandog et al. 2024). Mechanistic murine studies suggest that butyrate enhances CD8^+^ T cell memory and effector function through HDAC inhibition and increased mechanistic target of rapamycin complex 1 (mTORC1) activity (Luu et al. 2021; McBride et al. 2023).

Critical caveats:

- Human data are correlational and retrospective. They cannot distinguish whether high Lachnospiraceae abundance is a cause or a consequence of a strong antitumor immune response.
- Confounding by diet, antibiotic use, and other microbiota members is substantial and not fully controlled.
- No prospective trial has tested whether increasing Lachnospiraceae (via probiotics, fecal microbiota transplantation [FMT], or dietary fiber) improves ICI response rates.
- The reported associations do not demonstrate causality, and effect sizes are modest (e.g., odds ratios of 1.5–2.0 for response).

Evidence grading (Lachnospiraceae → antitumor immunity):

- Moderate in murine models (several independent studies)
- Low in humans (correlational only; no interventional data)

Therefore, although the hypothesis is promising, it is premature to recommend Lachnospiraceae‐based interventions as adjuncts to cancer immunotherapy outside of clinical trials.

### 3.4 Integrated Synthesis: Mechanisms Across Disease Contexts

Butyrate emerges as a central hub across multiple mechanistic layers in this framework, yet its pleiotropic effects raise critical questions about context‐dependent outcomes (Table 5). Butyrate simultaneously promotes Treg/TFR differentiation via HDAC inhibition and Foxp3 acetylation (Furusawa et al. 2013; Campbell et al. 2013; Smith et al. 2013), suppresses IL‐17‐producing γδ T cells through HDAC inhibition (as demonstrated for propionate, a related SCFA) (Dupraz et al. 2021) and Th17 polarization through GPCR (GPR41/43) engagement (Haghikia et al. 2015; Golpour et al. 2023), reinforces intestinal barrier integrity by upregulating ZO‐1, occludin, and claudin‐1 (Wang et al. 2012; Kelly et al. 2015), drives M2 macrophage polarization via NF‐κB inhibition (Chang et al. 2014; Huang et al. 2022), and enhances CD8^+^ T cell effector function through HDAC/mTORC1 signaling (Luu et al. 2021; Ji and Hu 2019; Gaskarth et al. 2025). This convergence suggests that butyrate acts not merely as a single‐pathway agonist but as a global rheostat that calibrates both innate and adaptive immunity. However, the strength of preclinical evidence (strong to moderate) contrasts sharply with low or very low human evidence for most butyrate‐related mechanisms, highlighting a major translational gap. Factors such as inter‐individual variation in butyrate production, colonic absorption efficiency, and tissue‐specific HDAC isoform expression may explain why murine observations do not reliably predict human responses (Smith et al. 2013; Kelly et al. 2015). Moreover, the opposing effects of butyrate on Treg (pro‐tolerogenic) versus CD8^+^ T cells (pro‐inflammatory/antitumor) illustrate a potential therapeutic paradox: systemic butyrate elevation might favorably dampen autoimmunity in the gut, yet inadvertently impair antitumor CD8^+^ immunity unless properly timed or locally delivered. Therefore, future human studies must move beyond correlational fecal SCFA measurements toward pharmacokinetic profiling of butyrate in plasma and target tissues, coupled with longitudinal immune monitoring, to decipher whether the net effect of butyrate is protective, detrimental, or strictly disease‐contextual.

**TABLE 5:** Unified mechanistic framework—Cross‐reference to disease applications.

Mechanism	Key molecular mediators	Evidence level (preclinical)	Evidence level (human)	Relevant disease contexts
HDAC inhibition → Treg/TFR differentiation Furusawa et al. (2013), Campbell et al. (2013), Smith et al. (2013)	Butyrate, Foxp3 acetylation	Strong	Low	Autoimmune (RA, IBD)
GPCR activation (GPR41/43/109A) → Th17 suppression Haghikia et al. (2015), Golpour et al. (2023)	Butyrate, propionate	Moderate	Very low	Autoimmune, metabolic
Flagellin‐TLR5 → DC‐mediated Treg induction Atarashi et al. (2013)	Flagellin, CD103^+^ DCs	Moderate (strain‐specific)	None	Autoimmune, cancer
EPS → immune shielding Round and Mazmanian (2010)	EPS (not limited in Lachnospiraceae)	Hypothetical	None	Autoimmune
Bile acid metabolites → FXR/TGR5 activation Makishima et al. (1999), Hang et al. (2019), Partney and Yissachar (2022)	DCA, LCA, 3‐oxoLCA, isoalloLCA	Moderate (other genera)	Low	Metabolic, cancer
Tight junction upregulation → barrier integrity Wang et al. (2012), Kelly et al. (2015)	Butyrate, ZO‐1, occludin, claudin‐1	Strong	Low	All inflammatory diseases
M2 macrophage polarization Chang et al. (2014), Huang et al. (2022)	Butyrate, NF‐κB inhibition	Moderate	Very low	Metabolic
CD8^+^ T cell effector enhancement Luu et al. (2021), Ji and Hu (2019), Gaskarth et al. (2025)	Butyrate, HDAC inhibition, mTORC1	Moderate	Low (correlational)	Cancer immunotherapy
Intestinal γδ T cells Dupraz et al. (2021)	Propionate repress IL‐17‐ and IL‐22; HDAC inhibition	Strong	Strong (correlational)	IBD

Abbreviations: DC, dendritic cell; DCA, deoxycholic acid; EPS, extracellular polysaccharides; FXR, farnesoid X receptor; GPCR, G‐protein‐coupled receptor; IBD, inflammatory bowel disease; mTORC1, mechanistic target of rapamycin complex 1; TLR5, toll‐like receptor 5; ZO‐1, zonula occludens‐1.

Non‐butyrate mechanisms—particularly bile acid metabolites and flagellin‐TLR5 signaling—remain underexplored but offer promising disease‐specific opportunities that may circumvent the pleiotropic uncertainty of butyrate (Table 5). Secondary bile acids such as DCA, LCA, 3‐oxoLCA, and isoalloLCA activate FXR and TGR5, with emerging evidence linking them to metabolic regulation and cancer immune surveillance (Makishima et al. 1999; Hang et al. 2019; Partney and Yissachar 2022). However, most supportive data originate from other bacterial genera (e.g., Clostridium and Bacteroides), and direct characterization within Lachnospiraceae is conspicuously absent, as reflected by the “hypothetical” evidence level for EPS‐mediated immune shielding (Round and Mazmanian 2010). Flagellin‐TLR5‐driven CD103^+^ DC‐mediated Treg induction (Atarashi et al. 2013) is another mechanistically attractive pathway, but its strict strain‐specificity and complete lack of human evidence relegate it to a mechanistic candidate rather than a validated target. These gaps are not merely academic; they define actionable priorities for translational research. For instance, the FXR/TGR5 axis already has small‐molecule modulators in clinical development for metabolic diseases, offering a faster repurposing route if microbiota‐derived bile acid metabolites can be reliably correlated with human disease endpoints. Conversely, the EPS immune shielding hypothesis (Round and Mazmanian 2010) remains purely theoretical in Lachnospiraceae and requires foundational work, including EPS structure elucidation, purification, and functional assays in humanized mouse models, before any clinical inference can be made. Collectively, although butyrate dominates the current evidence landscape, the strategic diversification toward bile acid metabolites and flagellin‐based pathways may ultimately yield more disease‐tailored interventions—particularly for metabolic diseases and cancer immunotherapy, where butyrate's dual effects on Treg and CD8^+^ T cells present a clear risk‐benefit trade‐off that non‐butyrate pathways could help resolve.

Key takeaway: The mechanistic framework presented in Section 3 provides the biological rationale for Lachnospiraceae‐mediated immunomodulation. However, as shown in the evidence grading above, the translation of these mechanisms to human disease contexts remains largely unproven. The following sections build upon this framework by examining dietary modulation strategies, disease‐specific applications, and translational barriers—while consistently referencing back to the mechanistic evidence and its limitations.

## 4 Application Evaluation of Lachnospiraceae in Disease Treatment—A Critical Appraisal of Evidence

Lachnospiraceae are among the most abundant anaerobic bacterial groups in the human gut, and their roles in maintaining host microecological homeostasis have been extensively studied in preclinical models. Recent years have seen a surge of interest in their therapeutic potential for modulating immune responses, regulating metabolism, enhancing antitumor immunity, and combating infections. However, a critical examination reveals that the majority of evidence remains preclinical, with human data largely correlational and subject to significant confounding. This section evaluates the mechanisms of action and therapeutic applications across four major disease areas—autoimmune/inflammatory diseases, metabolic disorders, cancer immunotherapy, and infectious diseases—while explicitly distinguishing animal/in vitro findings from human clinical evidence, highlighting inconsistencies, and identifying methodological limitations. Table 5 provides an overview of key studies and their evidence levels. A summary of the evidence strength across disease areas is presented in Table 6.

**TABLE 6:** Evidence strength summary for Lachnospiraceae in major disease areas.

Disease area	Preclinical evidence	Human observational evidence	Human interventional evidence	Causal evidence established?	Key knowledge gaps
Autoimmune/Inflammatory (RA, IBD) Bhutta et al. (2024), Barendregt et al. (2025)	Moderate–Strong	Low (small cohorts, confounding)	Very low (pilot studies only)	No	No RCTs with defined strains; strain‐specific effects unknown
Metabolic (T2D, obesity) Chávez‐Carbajal et al. (2019), Telle‐Hansen et al. (2022), Ray et al. (2025)	Moderate–Strong	Moderate (consistent associations)	Low (small trials, mixed formulations)	No	Contribution of Lachnospiraceae vs. diet not isolated
Cancer immunotherapy Dravillas et al. (2025), Charalambous et al. (2025), Mattavelli et al. (2026)	Moderate	Moderate (retrospective cohorts)	None (ongoing trials only)	No	Causality vs. correlation unresolved
Infectious diseases Xiao et al. (2025)	Moderate	None	None	No	Safety in immunocompromised patients unknown

Abbreviations: IBD, inflammatory bowel disease; RCT, randomized controlled trial; T2D, Type 2 diabetes.

### 4.1 Autoimmune and Inflammatory Diseases

#### 4.1.1 Modulation of Treg/Th17 Balance: Preclinical Mechanisms

Lachnospiraceae regulate host immune homeostasis primarily through the production of SCFAs via dietary fiber fermentation. Butyrate, the most extensively studied SCFA, acts through two principal pathways: (i) inhibition of histone deacetylases (HDACs), which enhances acetylation of histones H3 and H4 at the Foxp3 gene promoter and promotes the differentiation of regulatory T cells (Tregs) and follicular regulatory T cells (TFRs); and (ii) activation of GPCR(GPR41, GPR43, and GPR109A), which suppresses pro‐inflammatory Th17 cell generation and restores the Treg/Th17 balance—a homeostasis frequently disrupted in rheumatoid arthritis (RA) and IBD (Takahashi et al. 2020; McBride et al. 2023; Saadh et al. 2025). These mechanistic pathways are detailed in Section 3.1, and the subsequent paragraphs summarize key preclinical studies.

In a CIA mouse model, oral administration of microbial‐derived butyrate significantly attenuated synovial hyperplasia and joint destruction. Adoptive transfer of butyrate‐treated iTFR cells suppressed type II collagen‐specific autoantibodies and alleviated disease symptoms (Takahashi et al. 2020). E. rectale, a representative butyrate‐producing Lachnospiraceae species, has been shown to reduce ROS and nitric oxide levels in stressed intestinal epithelial cells via secreted metabolites, while downregulating genes associated with cellular stress and immune activation, thereby reinforcing its anti‐inflammatory potential (Vacca et al. 2020). In IL‐10‐deficient mice (a spontaneous colitis model), E. rectale supplementation attenuated local intestinal inflammation and reduced oxidative stress markers, enhancing overall gut microenvironment stability (Vacca et al. 2020).

Several caveats temper the translation of these preclinical findings to human autoimmune and inflammatory diseases. First, the supraphysiological butyrate concentrations (0.5–5 mM) used in most in vitro assays exceed the estimated physiological levels in the lamina propria (0.1–1 mM), raising concerns that observed immunomodulatory effects may be artifactually amplified or qualitatively distinct in vivo. Second, substantial species differences exist in SCFA receptor expression and downstream signaling between murine and human immune cells, rendering direct extrapolation unreliable. Third, most preclinical studies employ undefined Lachnospiraceae‐enriched consortia rather than axenic strains, precluding attribution of effects to specific taxa. The specific limitations of evidence in this disease context are summarized in the integrated critical appraisal framework presented in Section 6.1, along with cross‐cutting methodological concerns. Future work must prioritize (i) dose–response experiments using physiological SCFA concentrations relevant to human colonic tissue (Boets et al. 2016); (ii) comparative human–mouse immune cell assays to identify conserved versus species‐specific pathways (Zhiwei et al. 2016); and (iii) rigorous strain‐level characterization with isogenic mutants to establish causal links between bacterial genes and host immune outcomes (Kinnebrew et al. 2012). Only by addressing these caveats can the field move from correlation toward evidence‐based applications.

#### 4.1.2 Clinical Evidence in RA and IBD—Pilot Studies Only

Early‐phase clinical trials have begun to explore gut microecological modulation as a therapeutic strategy, but the evidence remains preliminary (Baydoun et al. 2025). In small cohorts of RA and IBD patients (*n* = 10–30 per study), pilot studies involving probiotic formulations containing Lachnospiraceae or FMT from healthy donors have reported decreased serum pro‐inflammatory cytokine levels (IL‐6, TNF‐α, and IL‐17) and improvements in clinical disease activity indices (Takahashi et al. 2020; Xu et al. 2024; Wang et al. 2026). However, these studies are limited by (i) small sample sizes; (ii) open‐label designs (lack of blinding); (iii) concomitant use of immunosuppressive medications that confound results; (iv) the absence of strain‐level characterization of the administered bacteria; and (v) short follow‐up periods (typically 4–12 weeks). Importantly, no RCT has been published that specifically tests a defined Lachnospiraceae strain in RA or IBD patients—the only RCT‐tested Lachnospiraceae strain (*A. soehngenii*) has been studied exclusively in metabolic disease populations. Therefore, claims of “promising efficacy” must be viewed with caution. Future research requires multicenter, double‐blind, placebo‐controlled RCTs with strain‐level characterization, standardized outcome measures (e.g., endoscopic remission in IBD and ACR50 in RA), and long‐term safety follow‐up.

Critical appraisal of human evidence—the specific limitations of evidence in this disease context are summarized in the integrated critical appraisal framework presented in Section 6.1, along with cross‐cutting methodological concerns.

Key unanswered questions: Do Lachnospiraceae play a causal, permissive, or merely bystander role in autoimmune disease pathophysiology? Can oral supplementation with a defined strain achieve sufficient colonic colonization to exert therapeutic effects in patients with chronic inflammation?

### 4.2 Metabolic Diseases (T2D and Obesity)

#### 4.2.1 Mechanisms of Metabolic Regulation—Evidence From Animal Models

In metabolic diseases, gut microbiota dysbiosis is closely linked to insulin resistance and chronic low‐grade inflammation in adipose tissue. Lachnospiraceae ferment dietary fiber to generate butyrate and propionate, which function as signaling molecules across multiple cell types and organs. Butyrate activates AMP‐activated protein kinase (AMPK) signaling in the liver and skeletal muscle, enhancing mitochondrial function and fatty acid oxidation, thereby improving energy metabolism and increasing insulin sensitivity (Grasset et al. 2022). Besides, Lachnospiraceae also regulates immune homeostasis primarily via SCFA‐mediated HDAC inhibition and GPCR activation (detailed in Section 3.1), promoting Treg/TFR differentiation and restoring the Treg/Th17 balance in autoimmune and inflammatory conditions (Grasset et al. 2022; Li et al. 2024).

In high‐fat diet‐induced obese mouse models, dietary interventions that increase Lachnospiraceae abundance (e.g., supplementation with inulin or resistant starch) elevate cecal butyrate levels, concomitant with improved glycemic control, enhanced insulin tolerance, and reduced endotoxemia (Baxter et al. 2019; Grasset et al. 2022). Mechanistic studies using Nlrp12‐deficient mice have shown that the absence of this innate immune receptor leads to reduced Lachnospiraceae abundance, decreased expression of SCFA synthesis enzymes, and heightened NF‐κB/MAPK activation, which exacerbates obesity‐associated metabolic inflammation (Lau and Dombrowski 2018). Supplementation with Lachnospiraceae or SCFAs reversed these effects, supporting a causal role in this specific mouse model (Figure 4C).

Critical appraisal—metabolic mechanisms: The limitations highlight a fundamental and often underestimated gap in metabolic microbiome research: the persistent inability to determine whether altered Lachnospiraceae abundance is a cause, a consequence, or merely a correlate of metabolic disease.

First, the overreliance on cross‐sectional study designs prevents the establishment of temporal causality. The reduction of SCFA‐producing Lachnospiraceae in T2D patients could equally be the result of hyperglycemia, insulin resistance, and other metabolic derangements reshaping the gut microbiota, rather than the cause.

Second, translating findings from animal models to clinical settings is fraught with uncertainty. Significant interspecies differences exist between mice and humans regarding gastrointestinal physiology, butyrate metabolism rates, absorption efficiency, and systemic bioavailability. Therefore, mechanisms validated in murine models—such as AMPK activation, GPR41/43 signaling, or HDAC inhibition—may not operate with the same magnitude, or even the same directionality, in humans.

Third, inadequate control for confounding factors is pervasive. Antidiabetic medications, particularly metformin, are well‐established modifiers of gut microbiota composition, whereas variables, such as diet, physical activity, and comorbidities, are often oversimplified in analyses. In the context of gestational diabetes mellitus (GDM), the association between butyrate and placental inflammation may also be driven by unmeasured confounders like maternal diet or medication use, rather than a direct causal effect (Huang et al. 2023).

To advance the field, future research must prioritize (i) prospective cohort studies with pre‐disease baseline sampling to establish temporality; (ii) adequately powered intervention trials that stratify for metformin use and other key confounders; and (iii) tissue‐level measurements (e.g., colonic biopsy SCFA quantification) instead of relying solely on fecal proxies. Until these methodological improvements are implemented, the causal role of Lachnospiraceae in metabolic disease will remain unproven.

#### 4.2.2 Microecological Intervention Strategies—Human Evidence Is Weak

Modifying dietary structure (e.g., increasing dietary fiber to ≥30 g/day) has been shown to increase Lachnospiraceae relative abundance and fecal SCFA concentrations in some human cohorts, but responses are highly individual (Baxter et al. 2019). A few small intervention trials have tested probiotic–prebiotic synbiotics containing Roseburia or Eubacterium strains; these report modest improvements in HbA1c, homeostatic model assessment of insulin resistance (HOMA‐IR), and body weight. However, most of these trials (i) lack a placebo control; (ii) use mixed probiotic formulations (making it impossible to attribute effects to Lachnospiraceae alone); (iii) have small sample sizes (*n* < 50); and (iv) do not perform strain‐level characterization or measure SCFA production in situ (Kim et al. 2022).

Collectively, High‐fiber diets are safe, inexpensive, and recommended for metabolic health. However, the specific contribution of Lachnospiraceae to the observed benefits remains unproven (Liu et al. 2026). Personalized approaches based on baseline microbiome profiling may improve response rates, but this hypothesis requires prospective validation.

##### 4.2.2.1 Critical Appraisal—Human Intervention Evidence

The specific limitations of evidence in this disease context are summarized in the integrated critical appraisal framework presented in Section 6.1, along with cross‐cutting methodological concerns.

Dose‐finding data are entirely absent—optimal strain, cell dose, frequency, and duration remain unknown. Without such pharmacokinetic and pharmacodynamic parameters, Phase 3 trial design is arbitrary, and negative results from under‐ or overdosed studies would be uninterpretable. Substantial inter‐individual variability in SCFA production and glycemic response—driven by baseline microbiome, genetics, diet, and medications—further argues against a one‐size‐fits‐all approach, yet no validated biomarkers exist for patient stratification.

Surrogate endpoints (HbA1c and HOMA‐IR) dominate, yet they do not guarantee clinically meaningful benefits such as reduced cardiovascular events or mortality—effect sizes often diminish when harder endpoints are assessed. Sustainability remains untested; most studies follow patients for weeks, not months or years, with no evidence of durable engraftment or metabolic benefit beyond the intervention period.

Future research must prioritize: Key research priorities for this disease context are detailed in the integrated framework presented in Section 6.4.

### 4.3 Cancer Immunotherapy

#### 4.3.1 Enhancement of Antitumor Immune Responses—Preclinical and Mechanistic Evidence

The gut microbiome has emerged as a key modulator of response to ICIs (Cai et al. 2025). Meta‐analyses of multiple cohorts show that bacteria associated with favorable response are concentrated within the Lachnospiraceae/Ruminococcaceae families of Firmicutes. Retrospective analyses of patients with melanoma, NSCLC, and renal cell carcinoma have consistently shown that responders to anti‐PD‐1/PD‐L1 therapy have higher baseline abundance of Lachnospiraceae, particularly the Lachnospiraceae_UCG_010 group, compared to non‐responders. The BiomeOne algorithm, incorporating gut microbiota features, demonstrated 78.6% sensitivity in predicting clinical benefit in a multicenter cohort.

Mechanistically, butyrate produced by Lachnospiraceae enhances CD8^+^ T cell memory and effector phenotypes through HDAC inhibition and GPCR‐mediated signaling. These effects increase intracellular mTORC1 activity, promote cell proliferation, and enhance secretion of cytotoxic molecules (perforin and granzyme B) and pro‐inflammatory cytokines (IFN‐γ and TNF‐α). In the tumor microenvironment of colorectal cancer, high Immunoscore—reflecting lymphocyte infiltration—was associated with high abundance of Lachnospiraceae in patient microbiomes, suggesting a potential link between these bacteria and antitumor immune surveillance (Hexun et al. 2023).

A particularly notable mechanistic finding has emerged from longitudinal studies: patients with complete responses to anti‐PD‐1 therapy exhibit stable gut microbiota functions during treatment, with a key role for Lachnospiraceae‐carried flagellin genes. Researchers identified MHC class I‐restricted peptides derived from flagellin‐related genes of Lachnospiraceae (FLach) that share structural homology with tumor‐associated antigens. FLach‐reactive CD8^+^ T cells were detectable in complete responders before ICI therapy, and FLach peptides have been shown to improve antitumor immunity in experimental models. This antigen mimicry mechanism provides a plausible causal link between Lachnospiraceae and ICI response that extends beyond the well‐characterized SCFA‐mediated immunomodulation. However, these findings remain primarily observational; the causal role of FLach peptides in human antitumor responses requires prospective validation.

In murine solid tumor models (e.g., melanoma and colon carcinoma), FMT from ICI‐responsive patients—which is enriched in Lachnospiraceae—increased CD8^+^ T cell infiltration into tumors and improved ICI efficacy.

Critical limitations of the human evidence: The human evidence linking Lachnospiraceae to ICI response is fundamentally correlational and confounded, creating a stark disconnect from promising preclinical findings. Although murine models show that FMT from responders (enriched in Lachnospiraceae) enhances CD8^+^ T cell infiltration and ICI efficacy, all human studies are retrospective or cross‐sectional—unable to establish whether high Lachnospiraceae abundance drives response, reflects a favorable tumor microenvironment, or merely marks overall health. This directionality problem is compounded by inadequate control of confounders, notably antibiotic use and dietary patterns, which independently affect both microbiota and immune function.

Attribution is particularly challenging: positive correlations with Lachnospiraceae do not account for co‐varying taxa such as Faecalibacterium and Ruminococcus, which may independently contribute to antitumor immunity. The unique contribution of Lachnospiraceae cannot be isolated from correlation‐based data, fundamentally undermining causal claims. Moreover, the mechanistically attractive finding that FLach activate CD8^+^ T cells derives from a single cohort of only 23 patients—a small sample that demands independent validation in larger, multicenter cohorts before acceptance.

Collectively, no human study has yet demonstrated that increasing Lachnospiraceae improves ICI response rates. FMT–ICI trials provide proof‐of‐concept for microbiome modulation but do not establish a specific causal role for this family. Key research priorities for this disease context are detailed in the integrated framework presented in Section 6.4.

#### 4.3.2 Clinical Trial Evidence—Emerging but Hypothesis‐Generating

Contrary to the earlier statement that no clinical results are available, multiple early‐phase clinical studies have now reported preliminary activity for FMT combined with ICIs. These studies represent important proof‐of‐concept but must be interpreted with caution.

MIMic trial (NCT03772899): In a Phase 1 multicenter trial, 20 patients with treatment‐naïve advanced melanoma received a single oral FMT from healthy donors followed by anti‐PD‐1 therapy. The objective response rate (ORR) was 65%, with a median progression‐free survival (mPFS) of 29.6 months and median overall survival (mOS) of 52.8 months at >40 months follow‐up. Treatment was safe, with no FMT‐related serious adverse events. However, as a single‐arm Phase 1 trial with a small sample size (*n* = 20), results are hypothesis‐generating rather than definitive (Hadi et al. 2025).

PERFORM trial (NCT04163289): In a Phase 1 trial of 20 patients with treatment‐naïve metastatic renal cell carcinoma, healthy donor FMT (d‐FMT) (LND101) combined with ICI‐based regimens achieved an ORR of 50% (9/18 evaluable patients), including two complete responses (11%). The safety endpoint was met, with no serious FMT‐related toxicities. Notably, most treatment responders did not develop Grade 3 or higher immune‐related adverse events. Alpha diversity improvement and durable engraftment of taxa and metabolic functions associated with anti‐inflammatory properties correlated with reduced toxicity and improved response (Fernandes et al. 2026).

TACITO trial (NCT04758507): In a randomized, double‐blind, placebo‐controlled Phase 2a trial of 45 treatment‐naïve metastatic renal cell carcinoma patients, d‐FMT from complete ICI responders combined with pembrolizumab + axitinib did not meet the primary endpoint of 12‐month progression‐free survival (PFS) (70% vs. 41% for d‐FMT vs. placebo, *p* = 0.053). However, secondary endpoints showed significantly improved median PFS (24.0 vs. 9.0 months; HR = 0.50, *p* = 0.035) and ORR (52% vs. 32%). Safety was comparable between arms, with no unexpected toxicities. Microbiome analysis confirmed donor strain engraftment, increased α‐diversity, and greater β‐diversity shifts in d‐FMT recipients, with acquisition/loss of specific strains (not total engraftment) associated with the primary endpoint. Findings support safety and potential efficacy of selected d‐FMT to enhance ICI‐based treatment in mRCC, warranting further investigation (Porcari et al. 2026).

FMT in ICI‐refractory gastric cancer: A Phase 1 study of healthy d‐FMT combined with nivolumab in 10 patients with ICI‐refractory microsatellite‐stable gastric cancer demonstrated feasibility and safety, with ORR 20% and disease control rate 40%. Clinical benefit was associated with colonization of donor‐derived immunogenic microbes and activated peripheral immune populations (Zhang et al. 2026).

Meta‐analysis evidence: A recent systematic review and meta‐analysis of 36 studies (*n* = 2746) evaluating microbiome‐modulating strategies (MMS) in cancer patients receiving ICIs found a pooled ORR of 40% (95% CI: 31%–49%). However, the authors explicitly noted that these findings are non‐comparative and confounded by study differences, with high heterogeneity (*I* ^2^ = 63.4%, *p* = 0.0003), and emphasized the urgent need for large, biomarker‐driven RCTs (Thu et al. 2026).

Critical appraisal of FMT evidence: The specific limitations of evidence in this disease context are summarized in the integrated critical appraisal framework presented in Section 6.1, along with cross‐cutting methodological concerns.

Critical takeaway: The evidence for Lachnospiraceae in FMT‐ICI studies is low (Phase 1 single‐arm open‐label trials, n = 10–20, with no concurrent controls; Lachnospiraceae‐specific effects cannot be isolated). The accumulating FMT‐ICI trial data provide important proof‐of‐concept that microbiome modulation may enhance ICI efficacy (Bhutiani and Wargo et al. 2022; Elkrief et al. 2025; Han et al. 2026; Robinson et al. 2023). However, these studies do not establish a specific causal role for Lachnospiraceae. The unique contribution of Lachnospiraceae—as opposed to the dozens or hundreds of other bacterial taxa co‐transplanted in FMT—remains entirely unknown. At present, there is no evidence to support the clinical use of Lachnospiraceae supplementation as an adjunct to cancer immunotherapy outside of investigational settings.

#### 4.3.3 Clinical Trial Evidence—Preliminary But Inconclusive

Several early‐phase trials are ongoing (e.g., NCT04138953 and NCT04577799) that combine FMT from ICI‐responsive donors with checkpoint inhibitors in melanoma patients. Preliminary results from a Phase 1 trial (NCT04138953) reported that FMT plus pembrolizumab was feasible and showed some antitumor activity in a subset of patients, but the specific contribution of Lachnospiraceae was not assessed (Routy et al. 2023). Critically, these studies do not establish a causal role for Lachnospiraceae. At present, there is no evidence to support the clinical use of Lachnospiraceae supplementation as an adjunct to cancer immunotherapy. Patients should not seek such interventions outside of clinical trials.

Critical appraisal—the evidence linking Lachnospiraceae to enhanced ICI efficacy remains almost entirely correlational, with no RCTs testing defined Lachnospiraceae strains in cancer patients—the only RCT‐tested strain (*A. soehngenii*) has been studied exclusively in metabolic disease populations. This represents a fundamental gap that leaves effect size and causality in the ICI context unknown. FMT studies, which show highly variable responses (20%–65%), cannot attribute benefits specifically to Lachnospiraceae; these bacteria may be passive markers of a healthy microbiome rather than active drivers. Host genetics, tumor burden, and prior treatments likely outweigh any microbial contribution, suggesting that even if Lachnospiraceae play a role, the effect is probably modest and subgroup‐specific. Published data come from narrow, selected cohorts (e.g., treatment‐naïve melanoma) and are not generalizable to other cancers or heavily pretreated patients. Safety remains unexplored—the impact on ICI‐related colitis or pneumonitis is unknown, and butyrate's dual inflammatory/immunostimulatory properties warrant caution (Zhang et al. 2016). Key research priorities for this disease context are detailed in the integrated framework presented in Section 6.4.

### 4.4 Prevention and Treatment of Infectious Diseases

#### 4.4.1 Mechanisms of Pathogen Inhibition—In Vitro and Animal Evidence

Lachnospiraceae contribute to colonization resistance against enteric pathogens through multiple mechanisms. First, the fermentative production of butyrate and other SCFAs lowers intestinal luminal pH (to pH do–6.0), which directly inhibits the growth of acid‐sensitive pathogens such as Salmonella enterica and Escherichia coli (Vacca et al. 2020). Second, certain Lachnospiraceae strains secrete bacteriocins or other antimicrobial peptides that antagonize competitors. Third, they occupy adhesion sites on the intestinal epithelium, preventing pathogen attachment.

In a mouse model of C. difficile infection, replacing insoluble fiber with pectin in the diet significantly increased the abundance of Lachnospiraceae (especially L. pectinoschiza) and elevated cecal butyrate and propionate concentrations. This was accompanied by activation of the aryl hydrocarbon receptor (AhR) pathway, restoration of tight‐junction protein expression (ZO‐1, occludin), reduced mucosal LPS translocation, decreased systemic inflammatory markers, and amelioration of histological damage (Wu et al. 2022). In vitro experiments with E. rectale cell‐free supernatants reduced ROS production and downregulated stress‐related genes in intestinal epithelial cells, indicating a direct cytoprotective effect (Vacca et al. 2020).

##### 4.4.1.1 Critical Appraisal of Lachnospiraceae in Infectious Disease Prevention and Treatment

The specific limitations of evidence in this disease context are summarized in the integrated critical appraisal framework presented in Section 6.1, along with cross‐cutting methodological concerns.

#### 4.4.2 Bacterial Transplantation and Engineered Strains—Preclinical Only

FMT from healthy donors is an established treatment for recurrent *C. difficile* infection. Successful FMT is associated with restoration of Lachnospiraceae and other butyrate‐producing bacteria, but the specific contribution of Lachnospiraceae has not been isolated. Engineered Lachnospiraceae strains that overexpress antimicrobial peptides or butyrate synthesis genes remain at the proof‐of‐concept stage in animal models (Wang et al. 2024; Yang et al. 2024). Regulatory and safety hurdles for live engineered bacteria are substantial; clinical translation is likely >5 years away and remains speculative.

### 4.5 Integrated Critical Synthesis Across Disease Areas

Several cross‐cutting themes emerge from the disease‐specific evaluations above:

- The “Black Box” Problem: Nearly all human studies rely on 16S rRNA sequencing, providing only genus‐ or family‐level resolution. Given that metabolic and immunomodulatory functions are strain‐specific, family‐level conclusions are intrinsically unreliable—a fundamental limitation undermining the interpretability of most correlational studies.
- The Causality Chasm: Substantial correlational evidence links Lachnospiraceae abundance to disease states, but correlation does not imply causation. Confounders—diet, medication, disease duration, comorbidities, and lifestyle—are rarely adequately controlled. The few Mendelian randomization studies (e.g., Ren et al. 2025; Wang and Zheng 2025; Vacca et al. 2020) have yielded inconsistent results, underscoring the uncertainty.
- The Missing Clinical Trial Evidence: No RCT has tested a defined Lachnospiraceae strain in any disease, with the sole exception of A. soehngenii in metabolic disease (glycemic outcomes only). This absence of RCTs for the vast majority of strains represents the single most important barrier to clinical translation.
- The Strain‐Specificity Imperative: The literature often treats “Lachnospiraceae” as a homogeneous group with predictable functions. This is scientifically unjustified: distinct genera (Roseburia, Blautia, Eubacterium, Coprococcus, and Anaerobutyricum) have different metabolic capacities, and even strains of the same species can differ in SCFA production, adhesion, and immunomodulation.
- The Gut‐Food Axis Complexity: Responses to dietary interventions depend on baseline microbiome composition, host genetics, and food matrix. Substantial inter‐individual variation in response to the same fiber challenges a “one‐size‐fits‐all” approach and highlights the need for personalized strategies.

## 5 Dietary Modulation of Lachnospiraceae: Fiber Types, Food Processing, and Matrix Effects

This subsection is central to the food science focus of the review. The composition and metabolic activity of Lachnospiraceae are primarily shaped by dietary fibers, but not all fibers are equal. The chemical structure, degree of polymerization, solubility, and physical encapsulation of a fiber determine which Lachnospiraceae taxa can degrade it and what SCFA profile is produced.

### 5.1 Fiber‐Specific Effects

- Inulin and FOS: These soluble, prebiotic fibers are rapidly fermented by Roseburia and Blautia species, yielding high acetate and butyrate levels (Zhang et al. 2024). In a human randomized controlled trial, inulin supplementation (16 g/day for 2 weeks) significantly increased Roseburia abundance and fecal butyrate (Vandeputte et al. 2017). However, tolerance varies, and high doses can cause bloating and flatulence.
- Pectin: This complex polysaccharide is degraded by L. pectinoschiza and certain Roseburia strains. Pectin fermentation produces acetate first, followed by delayed butyrate production via cross‐feeding with other bacteria. In a C. difficile mouse model, replacing cellulose with pectin increased Lachnospiraceae abundance, elevated cecal butyrate and propionate, reduced inflammatory markers, and improved epithelial barrier function (Wu et al. 2022). Human data are limited.
- Resistant starch Type 3 (RS Type 3): RS3 is a potent butyrogenic substrate. It is primarily fermented by E. rectale, R. intestinalis, and Anaerostipes species. The butyrate yield from RS is among the highest of all fibers, as demonstrated in in vitro fecal fermentation systems (Louis and Flint 2017). Few human RCTs have tested RS3 interventions with strain‐level monitoring.
- Arabinoxylan: Found in whole grains (wheat, rye, and barley). It is selectively utilized by Roseburia and Blautia species, producing acetate and butyrate. Whole grain wheat consumption (replacing refined wheat) for 6 weeks has been shown to increase Roseburia abundance in humans (Vanegas et al. 2017). Effect sizes are modest and highly variable.
- Fiber mixtures: Importantly, fiber blends often produce synergistic SCFA outputs exceeding the sum of individual components. This is due to cross‐feeding among primary degraders (e.g., Bifidobacterium) and secondary butyrate producers (Lachnospiraceae). For example, a mixture of inulin, RS3, and pectin produced higher butyrate than any single fiber in an in vitro gut model (Opperman et al. 2026). However, human validation is lacking.

Critical Caveat: Most human studies measure fecal SCFA concentrations, which poorly reflect colonic tissue levels. SCFA are rapidly absorbed, and fecal levels represent only the excess that escapes absorption. Stable isotope tracer studies that measure SCFA production in situ are urgently needed.

### 5.2 Food Processing Effects

Food processing methods (extrusion, cooking, grinding, and milling) alter fiber structure and accessibility. Extrusion, for example, can disrupt the physical encapsulation of fibers within the food matrix, making them more rapidly fermentable. However, very rapid fermentation may lead to SCFA production in the proximal colon rather than the distal colon, where butyrate is most needed for barrier function and immune regulation (Rivière et al. 2016; Smith et al. 2022). Fine milling of whole grains increases the surface area for enzymatic attack, increasing fermentability but also potentially accelerating glucose release, with implications for glycemic response. Particle size reduction generally increases fermentation rate but may reduce butyrate yield if substrates are too rapidly degraded and consumed by other bacteria before Lachnospiraceae can utilize them. Systematic studies examining the effects of processing on Lachnospiraceae‐mediated fermentation are scarce.

### 5.3 Food Matrix Effects

The same fiber type in different food matrices (e.g., isolated inulin vs. inulin‐enriched bread) can produce different SCFA profiles due to interactions with other macronutrients (fat and protein), anti‐nutritional factors (phytates and tannins), and physical structure. Whole foods typically result in slower, more sustained fermentation compared to isolated fibers. For example, whole oats (with intact β‐glucan matrix) produce a different SCFA profile than purified β‐glucan. This matrix effect is understudied and represents an important research gap for functional food design.

### 5.4 Cross‐Feeding Networks

Lachnospiraceae often act as secondary fermenters, utilizing breakdown products (e.g., lactate and acetate) generated by primary degraders such as Bifidobacterium and Bacteroides. Therefore, dietary interventions that include both rapidly fermented fibers (to support primary degraders) and slowly fermented fibers (to sustain secondary fermenters) can maximize SCFA output through synergistic cross‐feeding. Rational design of fiber mixtures is a promising strategy for functional food development, but most evidence comes from in vitro models; human validation is scarce (Opperman et al. 2026).

### 5.5 Knowledge Gaps in Dietary Modulation of Lachnospiraceae for Functional Food Development

The evidence base for dietary modulation of Lachnospiraceae is constrained by critical knowledge gaps that impede rational intervention design. Dose–response relationships remain entirely undefined, leaving no guidance on which fiber types or quantities effectively promote target taxa. Processing effects are poorly characterized, with little mechanistic insight into how food preparation alters fiber bioaccessibility and subsequent microbial utilization. Matrix effects—the influence of food structure and co‐ingested components—are severely understudied, representing a major barrier to developing effective functional foods. Individual variability is substantial, with baseline microbiome composition and host genetics known to influence outcomes, yet no validated predictive algorithms exist to personalize dietary recommendations. Finally, the common reliance on fecal SCFA measurements is problematic, as these reflect luminal concentrations rather than tissue‐level activity, undermining the translation of microbiome data to physiological benefit. Collectively, these gaps highlight that current dietary strategies targeting Lachnospiraceae remain largely empirical and underscore the urgent need for systematic, controlled human intervention studies that integrate detailed metabolic phenotyping and advanced analytics to establish causal, dose‐dependent, and person‐specific effects.

### 5.6 Summary and Research Priorities

On the basis of the critical appraisal above, we identify the following priority research areas:

- Strain‐level characterization: Future studies must move beyond 16S rRNA sequencing to metagenomic and culture‐based approaches that enable strain‐level resolution.
- Mechanistic human studies: Stable isotope tracer studies and colonic tissue sampling are needed to measure SCFA production and activity in situ in humans.
- RCTs with defined strains: The single most important gap is the absence of RCTs testing defined Lachnospiraceae strains in patient populations.
- Causal inference methods: Mendelian randomization, longitudinal sampling, and intervention studies with appropriate control groups are needed to establish causality.
- Food matrix and processing research: Systematic studies are needed to guide the development of functional foods that optimize Lachnospiraceae‐mediated health benefits.
- Safety monitoring: Long‐term safety data in diverse populations, including immunocompromised individuals, are essential before clinical translation.

### 5.7 Applications in Functional Foods and Next‐Generation Probiotics—Practical Realities

#### 5.7.1 Strain‐Specific Effects and Functional Heterogeneity—A Critical Summary

The Lachnospiraceae family comprises >60 genera and hundreds of species‐level phylogroups, with substantial functional heterogeneity. Generalizing from one genus to the whole family is scientifically unjustified. Table 2 summarizes evidence for representative taxa, explicitly grading evidence strength and highlighting limitations.

Collectively, strain‐level characterization—including whole‐genome sequencing (to screen for antibiotic resistance and virulence genes), in vitro safety assays (hemolysis and biogenic amine production), and functional immune assays (cytokine profiling and Treg induction)—is mandatory before any Lachnospiraceae‐based product can be considered for food or therapeutic use. With the sole exception of *A. soehngenii* CH106, which has received FDA GRAS status for food use only (GRN No. 1065), no other Lachnospiraceae strain has GRAS or QPS status. The GRAS status of *A. soehngenii* does not extend to therapeutic applications, nor does it imply safety or approvability of other strains.

#### 5.7.2 Technological Challenges in Food Matrices

Developing Lachnospiraceae‐containing functional foods or dietary supplements faces formidable technical hurdles (Figure 5):

- Oxygen sensitivity: Lachnospiraceae are generally obligate anaerobes (https://beta.bacdive.dsmz.de); for example, A. soehngenii is classified as a strict anaerobe, and strict anaerobes are typically incapable of growth at pO_2_ levels exceeding 0.5%. However, oxygen tolerance varies across genera and strains, and strain‐specific measurements are require. This presents challenges for large‐scale fermentation (requires anaerobic fermenters), downstream processing (lyophilization under inert gas), packaging (oxygen‐barrier films with oxygen scavengers), and storage (refrigeration or frozen conditions). Shelf‐life under ambient conditions is currently limited; reported stability varies substantially depending on formulation and storage conditions, with some preparations requiring refrigeration or frozen storage.
- Viability during shelf‐life: Probiotic products typically require >10^6^ CFU/g at the end of a 12–24 month shelf‐life. For anaerobes, this is extremely challenging. Even with lyophilization and cryoprotectants, substantial viability losses have been reported, although the magnitude varies with formulation and strain. Postbiotics (heat‐killed cells or cell components) and metabolite‐based preparations (purified SCFAs or fermentation supernatants) offer alternative approaches with different safety profiles. Importantly, under the ISAPP definition, only preparations containing inactivated microbial cells or their components qualify as postbiotics, purified metabolites without cellular biomass do not.
- Gastrointestinal transit survival: After ingestion, bacteria must survive gastric acid (pH 1.5–3.0 for 1–2 h), bile salts (1–20 mM), and digestive enzymes (pepsin, trypsin, and chymotrypsin). Unprotected cells are rapidly killed. Encapsulation technologies—including alginate‐chitosan microcapsules (50–300 µm diameter), lipid‐based enteric‐coated capsules (designed to remain intact until pH > 6.0 in the ileum), and multi‐layered hydrogel microspheres—can improve survival but add cost and complexity. Recent advances in “Express Microcolony” platforms (alginate hydrogels with stress‐relaxing properties) have demonstrated up to 10,000‐fold improved viability compared to free cells under simulated gastrointestinal conditions (Smith et al. 2022).
- Food matrix compatibility: Live Lachnospiraceae are incompatible with high‐heat processing (baking, pasteurization, and sterilization), high salt (>2%), low pH (<4.5), or the presence of preservatives (e.g., potassium sorbate). Therefore, the only feasible food formats are refrigerated products (yoghurt, fermented milk, and plant‐based drinks) or freeze‐dried powders that are reconstituted immediately before consumption.

**FIGURE 5:**
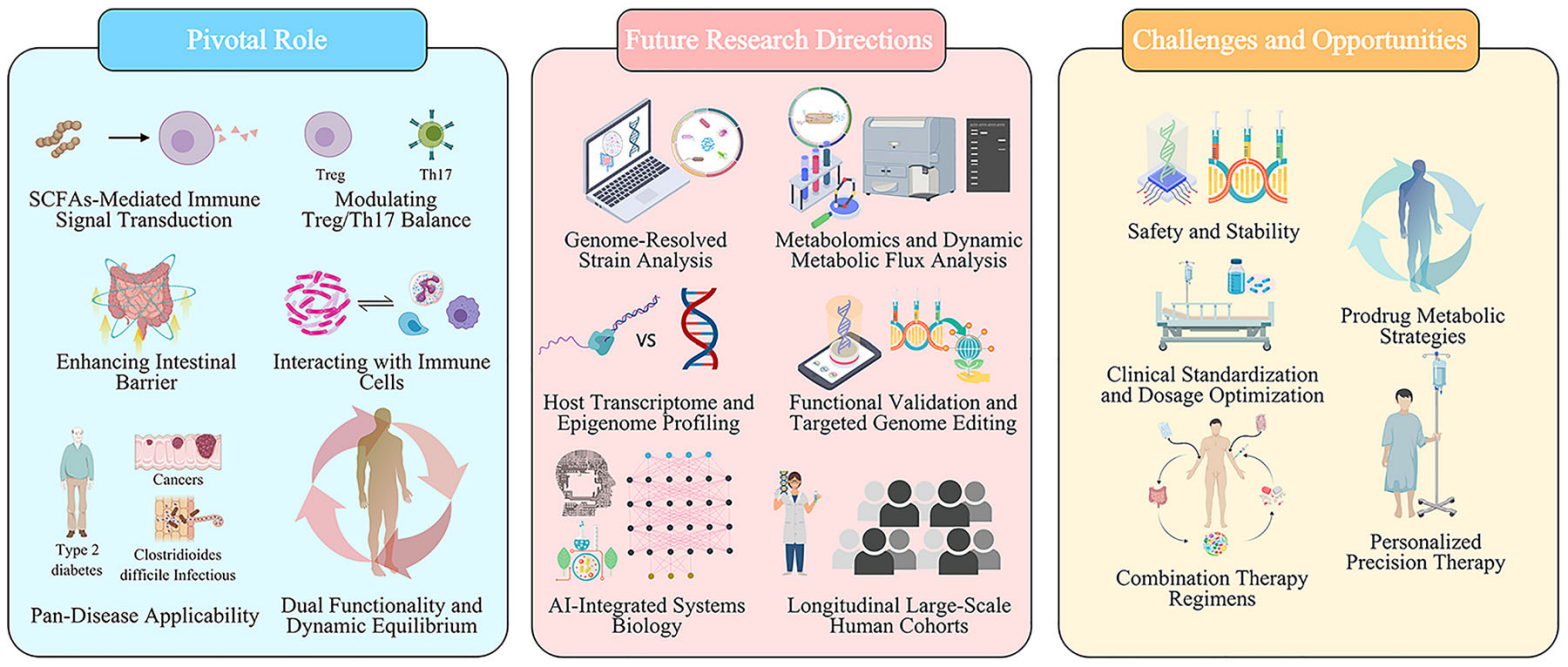
Comprehensive overview of pivotal roles, future research directions, and challenges/opportunities in Lachnospiraceae research. Pivotal Role: Summarizes the core mechanisms, including SCFAs‐mediated immune signal transduction, modulation of Treg/Th17 balance, enhancement of the intestinal barrier, and interactions with immune cells. It further highlights the broad pan‐disease applicability across conditions such as Type 2 diabetes, cancers, and Clostridioides difficile infections, emphasizing the dual functionality and dynamic equilibrium of these interventions. Future Research Directions: Outlines key scientific avenues requiring further exploration, including genome‐resolved strain analysis, metabolomics and dynamic metabolic flux analysis, host transcriptome and epigenome profiling, functional validation and targeted genome editing, AI‐integrated systems biology, and longitudinal large‐scale human cohort studies. Challenges and Opportunities: Highlights critical translational hurdles and emerging opportunities, encompassing safety and stability, prodrug metabolic strategies, clinical standardization coupled with dosage optimization, personalized precision therapy, and combination therapy regimens. SCFAs, short‐chain fatty acids.

### 5.8 Anaerobutyricum soehngenii: A Model Organism for Lachnospiraceae Translation

#### 5.8.1 Taxonomic Background and Discovery

A. soehngenii (formerly classified as E. hallii) is a gram‐positive, obligately anaerobic, butyrate‐producing bacterium within the family Lachnospiraceae (https://www.bacdive.org/strain/5429). The species was reclassified into the newly established genus Anaerobutyricum in 2018 based on polyphasic taxonomic analysis. The type strain L2‐7 was originally isolated from healthy infant feces. Importantly, A. soehngenii possesses glycerol/diol dehydratase activity, enabling it to utilize 1,2‐propanediol, which facilitates metabolic cross‐feeding with other gut bacteria that produce this intermediate.

#### 5.8.2 Regulatory Milestone—GRAS Status

In April 2023, A. soehngenii CH106 became the first and, to date, the only Lachnospiraceae strain to receive a “no questions” GRAS response from the FDA (GRN No. 1065). The intended use includes incorporation into various food matrices at a maximum level of 1.0 × 10^10^ total fluorescent units per serving. Importantly, this GRAS designation applies exclusively to food use; it does not constitute regulatory approval for therapeutic applications, nor does it imply that other Lachnospiraceae strains are safe. The intended use includes incorporation into various food matrices (e.g., sports drinks, breakfast cereals, yogurt, protein bars, and ice cream) at a maximum level of 1.0 × 10^10^ total fluorescent units per serving, as specified in the FDA GRAS notification (GRN No. 1065). It is critical to emphasize that this GRAS status applies exclusively to food use; it does not constitute regulatory approval for therapeutic applications, nor does it imply that other Lachnospiraceae strains are safe.

Important caveat: The GRAS status of A. soehngenii CH106 applies specifically to this strain and to food use, not to therapeutic indications. It does not imply that other Lachnospiraceae strains are safe or that regulatory approval is easily obtained. Each strain requires independent safety assessment, and the regulatory pathway for therapeutic applications (as a LBP) is substantially more demanding than for food ingredients.

#### 5.8.3 Clinical Evidence—RCTs

Contrary to the claim that no RCTs have been conducted with defined Lachnospiraceae strains, multiple RCTs have been published using A. soehngenii:

- A period of 14‐day RCT (*n* = 25): A randomized, double‐blind, placebo‐controlled trial in Dutch males with T2D on stable metformin therapy showed that A. soehngenii L2‐7 supplementation significantly improved glycemic variability and mean arterial blood pressure compared to placebo.
- A period of 3‐month RCT (*n* = 98): A multicenter, double‐blind, placebo‐controlled trial in prediabetic insulin‐resistant adults (Europe and US) demonstrated that daily oral supplementation with A. soehngenii CH106 (1 × 10^9^ viable cells/day):
  i. Reduced glycemic variability (1% reduction in coefficient of variation; *p* = 0.01)
  ii. Improved overall glycemic control (6% reduction in net glycemic action; *p* < 0.05)
  iii. Reduced HbA1c levels (1 mmol/mol reduction; *p* < 0.05, including washout period)
  iv. Lowered diastolic blood pressure (3 mmHg reduction; *p* < 0.05)

The study product was well‐tolerated, and no serious adverse events were reported. Notably, US participants (who had more severe prediabetic states and lower baseline abundance of Bifidobacterium, Coprococcus, and Ruminococcus) showed more pronounced responses.

Critical note: The RCT evidence for *A. soehngenii* is strain‐specific and indication‐specific. It demonstrates efficacy for glycemic control in metabolic disease populations but does not provide evidence for (i) other Lachnospiraceae strains; (ii) other disease indications (cancer immunotherapy, autoimmune diseases, and infectious diseases); or (iii) therapeutic claims beyond the specific formulations and doses tested. Overgeneralization from *A. soehngenii* to the entire family remains a major pitfall.

#### 5.8.4 Critical Appraisal of the Evidence for A. soehngenii: A Paired Discussion of Strengths and Limitations

The body of evidence supporting A. soehngenii as a potential modulator of glycemic control presents a mixture of methodological rigor and unresolved uncertainties. Below, each key strength is considered alongside its most pertinent limitation, to provide a balanced perspective.

- Trial Design versus Sample Size: A major strength is the reliance on well‐designed randomized controlled trials (RCTs) that incorporate placebo control and double‐blinding, which minimizes bias and strengthens causal inference. However, this commendable methodological rigor is tempered by modest sample sizes, ranging from 25 to 98 participants per study. Such limited numbers reduce statistical power for detecting smaller effect sizes and limit the precision of subgroup analyses, meaning that even robustly designed trials may yield results that are not yet definitive.
- Consistency of Glycemic Effects versus Population Homogeneity: The observed effects on glycemic parameters—such as fasting glucose, insulin sensitivity, or postprandial responses—have been remarkably consistent across independent studies, which lends credibility to the strain's biological activity. Nevertheless, all published trials have been conducted exclusively in Western populations (predominantly European or North American). This narrow demographic base leaves open the question of whether the same benefits would be observed in other ethnic groups, who may differ in baseline gut microbiota composition, dietary habits, and genetic background, thereby limiting the generalizability of the findings.
- GRAS Status versus Regulatory Scope: The strain's GRAS status provides a regulatory precedent that facilitates its incorporation into food products and offers some reassurance regarding its basic safety profile. Yet this GRAS designation applies strictly to food use, not to therapeutic claims. Therefore, although the strain can be added to foods without pre‐market approval, using it as a drug‐like intervention for managing glycaemia would require a separate regulatory pathway, and GRAS status does not substitute for the rigorous evidence needed to support clinical indications.
- Short‐Term Safety versus Long‐Term and Immunocompromised Populations: No serious adverse events have been reported in the short‐term trials (typically lasting several weeks to a few months), which is encouraging for initial tolerability. However, safety data beyond 1 year of continuous use are completely absent, and there is no information on how A. soehngenii might affect immunocompromised individuals—such as those undergoing chemotherapy, transplant recipients, or patients with severe immunodeficiency. In these vulnerable groups, even commensal organisms can pose risks, and the current evidence does not allow any conclusions about their safety.
- Mechanistic Plausibility versus Established Causal Pathway: The proposed mechanism—production of butyrate and other SCFAs—is biologically plausible and supported by in vitro and animal studies, providing a coherent rationale for the observed glycemic improvements. Nonetheless, the causal chain from butyrate production to clinical outcomes in humans has not been fully established. It remains unclear whether the effects are directly mediated by butyrate, by other bacterial metabolites, or by indirect interactions with the host immune or endocrine systems. Until human mechanistic data are obtained, the link between the strain's activity and its clinical benefits remains inferential rather than proven.

In sum, the evidence for *A. soehngenii* is moderate (multiple RCTs with consistent glycemic effects, but modest sample sizes and limited population diversity preclude a strong rating). The strengths lie in trial quality, consistency, and safety signals, whereas the limitations—chiefly small samples, narrow populations, regulatory boundaries, lack of long‐term data, and incomplete mechanistic proof—highlight the need for larger, more diverse, and longer term studies with integrated mechanistic endpoints before therapeutic applications can be confidently advocated.

#### 5.8.5 Lessons for the Lachnospiraceae Field

The trajectory of A. soehngenii—from discovery and taxonomic reclassification, through preclinical testing and mechanistic studies, to GRAS status and RCT evidence—offers a model roadmap for translating other Lachnospiraceae strains. However, several cautionary points must be emphasized:

- Strain specificity: The success of A. soehngenii should not be overgeneralized. Other Lachnospiraceae strains may lack the same safety profile, metabolic capacity, or clinical efficacy.
- Resource intensity: The path from discovery to GRAS status and RCT evidence required substantial investment in culturomics, genomic characterization, good manufacturing practice (GMP)‐grade production, and regulatory documentation.
- Regulatory distinction: GRAS status for food use is not equivalent to regulatory approval for therapeutic use (which requires LBP designation and full clinical trial phases).
- The “model organism” trap: Using A. soehngenii as a model for the entire family risks reinforcing the very overgeneralization that the field should avoid.

### 5.9 Regulatory Barriers and Pathways

With the sole exception of *A. soehngenii* CH106 (which has GRAS status for food use only), no other Lachnospiraceae strain currently possesses GRAS or QPS status. This lack of regulatory recognition for the vast majority of strains represents a significant barrier to food use. Importantly, even for *A. soehngenii*, GRAS status applies only to food ingredients—not to therapeutic claims—and does not generalize to other strains. The regulatory pathway for a new probiotic strain requires:

- For GRAS (US): Either self‐affirmation (manufacturer assembles safety evidence) or submission of a GRAS notification to the FDA. The latter involves a 90‐day review period; if the FDA issues a “no questions” letter, the strain can be marketed as a food ingredient. Required data include taxonomic identification at strain level, history of safe use in food, the absence of antibiotic resistance genes and virulence factors (from whole‐genome sequencing), in vitro safety assays (hemolysis, gelatinase, and biogenic amine production), and 28‐day oral toxicity study in rodents (Spacova et al. 2023).
- For QPS (EU): European Food Safety Authority (EFSA) evaluates the taxonomic unit (species or genus). Strains placed on the QPS list do not require further case‐by‐case safety assessment. However, to date, no Lachnospiraceae species has been evaluated for QPS, largely because this family lacks a long history of safe use in foods or feeds. EFSA has explicitly stated that “many emerging microbial candidates, including most Lachnospiraceae strains, lack established safety status” (EFSA Panel on Biological Hazards (BIOHAZ) et al. 2017).

Given these hurdles, pragmatic near‐term alternatives include the development of postbiotics—preparations of inanimate microorganisms and/or their components (e.g., cell walls and surface structures) that confer health benefits—as well as metabolite‐based preparations (e.g., purified SCFAs or fermentation supernatants). Critically, the ISAPP definition requires that postbiotics contain inactivated microbial cells or cell components, with or without metabolites; purified metabolites without cellular biomass do not qualify as postbiotics (Salminen et al. 2021). Postbiotics are not subject to the stringent regulatory frameworks governing live microorganisms; they can be marketed as food ingredients with a simpler safety dossier (generally, they must be nontoxic and free of harmful contaminants). Advantages include no viability requirement, longer shelf‐life (12–24 months at ambient temperature), no risk of translocation or infection in immunocompromised hosts, and easier incorporation into a wide range of food matrices (baked goods, beverages, and snacks). A liposome‐microcapsule butyrate formulation (a postbiotic) has already shown clinical benefit in a small CD trial, with improved clinical activity scores and a measured 18% increase in fecal Lachnospiraceae NK4A136 (likely due to cross‐feeding).

#### 5.9.1 Metabolite‐Based Approaches (Fermentation Supernatants and Purified SCFAs)—Distinction From Postbiotics

Terminological clarification: According to the ISAPP 2021 consensus definition, a postbiotic is “a preparation of inanimate microorganisms and/or their components that confers a health benefit on the host” (Salminen et al. 2021; Vinderola et al. 2022). This definition requires that inactivated microbial cells or cell components be present, with or without metabolic end products. Critically, purified microbial metabolites (e.g., butyric acid and propionic acid) and cell‐free supernatants devoid of microbial biomass do not qualify as postbiotics (Salminen et al. 2021; Meena et al. 2025); such preparations should be described using existing chemical nomenclature or as “metabolite‐based preparations.” The ISAPP panel explicitly stated that “substantially purified metabolites in the absence of cellular biomass” fall outside the definition, and such preparations should instead be described using existing, clear chemical nomenclature (Salminen et al. 2021). This distinction has been further reinforced in a subsequent ISAPP‐affiliated FAQ publication, which addresses why microbially produced metabolites do not qualify as postbiotics (Meena et al. 2025; Swanson et al. 2020).

The distinction is not merely semantic: postbiotics retain the structural components of microorganisms (e.g., cell wall polysaccharides, pili, and surface proteins) that may contribute to immunomodulatory effects through host receptor interactions, whereas purified metabolites act through different (primarily biochemical) mechanisms (Salminen et al. 2021; Vinderola et al. 2023; Mishra et al. 2024). In this review, we therefore use the term “metabolite‐based preparations” or “fermentation‐derived metabolites” to describe SCFA‐rich fermentation supernatants, cell‐free broths, and purified metabolites, reserving the term “postbiotic” exclusively for preparations containing inanimate microbial biomass as defined by ISAPP (Balakrishna et al. 2026).

#### 5.9.2 Rationale for Metabolite‐Based Approaches

The production of fermentation‐derived metabolites, including SCFA‐rich supernatants and purified butyrate or propionate preparations, represents a potentially pragmatic pathway for delivering the bioactive metabolites of Lachnospiraceae without the regulatory and technical burdens associated with live bacterial cultures. These preparations can be produced by fermenting dietary fibers with selected Lachnospiraceae strains (e.g., E. rectale or R. intestinalis), followed by heat killing (e.g., 85°C for 15 min) or microfiltration to remove live cells. The resulting metabolite‐rich preparations can be incorporated into functional foods or used as ingredients in clinical nutrition products (Schluter et al. 2026; Balakrishna et al. 2026; Guglielmetti et al. 2025; Wei et al. 2024; Sanders et al. 2019). Notably, these metabolite‐based preparations—unlike postbiotics—do not contain microbial biomass and therefore do not meet the ISAPP definition of a postbiotic. They represent a distinct category of products that deliver specific bioactive compounds rather than the complex matrix of inactivated microbial cells.

An important distinction must be made between “postbiotics” (as defined by ISAPP) and simple metabolite mixtures: A postbiotic preparation containing heat‐killed Lachnospiraceae cells plus their fermentation metabolites would qualify under the ISAPP definition, as it contains inanimate microbial biomass. However, a filtered fermentation broth that contains only metabolites (SCFAs, organic acids, and vitamins) and no microbial cellular material does not qualify as a postbiotic—it is simply a metabolite‐containing preparation that should be described as such (Hill et al. 2014; Salminen et al. 2021; Meena et al. 2025; Emília 2024; Taspinar and Güzeler 2026).

#### 5.9.3 Critical Caveats and Limitations

Even if these prerequisites are met, metabolite‐based preparations do not contain live microorganisms and therefore cannot restore colonization or ecological functions of Lachnospiraceae. They represent a delivery vehicle for specific metabolites, not a replacement for live bacterial therapies. The biological activity of a complex fermentation supernatant cannot be assumed to replicate the effects of live bacteria, as the latter may have additional functions (e.g., niche occupation, cross‐feeding, immunomodulatory signaling via surface structures) that are absent in metabolite‐only preparations.

Key distinction—ISAPP definition of postbiotics versus metabolite preparations (Sanders et al. 2019; Salminen et al. 2021): The comparative framework reveals that postbiotics and metabolite‐based preparations, whereas both derived from microbial activity, are fundamentally distinct in composition, mechanism, and regulatory standing. Postbiotics contain inanimate microbial biomass (e.g., heat‐killed cells) alongside their structural components, enabling a dual mode of action that combines cell‐surface interactions (via microbe‐associated molecular patterns [MAMPs] and pili) with metabolite‐driven effects. In contrast, metabolite‐based preparations are chemically defined, containing only purified metabolites such as SCFAs or organic acids, and thus rely exclusively on receptor‐mediated or epigenetic pathways. This compositional divergence has practical implications: postbiotics may offer broader, multitargeted effects due to their complex matrix, but their heterogeneous nature complicates standardization and mechanistic attribution; metabolite‐based products are more amenable to precise dosing and quality control, yet they may lack the pleiotropic benefits of whole‐cell preparations. From a regulatory perspective, postbiotics can fall under multiple categories (foods, supplements, or drugs) depending on health claims, whereas metabolite‐based preparations are typically treated as food ingredients or isolated chemicals, which may streamline market entry but also constrain therapeutic positioning. Ultimately, the choice between these two approaches should be guided by the intended application—prophylactic functional foods might favor postbiotics, whereas targeted pharmacological interventions could benefit from well‐defined metabolite formulations—but both require rigorous strain‐specific and product‐specific validation to support efficacy and safety claims.

Important note: A fermentation supernatant that contains both microbial cell debris and metabolites qualifies as a postbiotic under the ISAPP definition, provided the preparation is characterized and the health benefit is demonstrated (Salminen et al. 2021). However, a supernatant that has been filtered to remove all cellular material does not meet the definition and should be termed a “fermentation‐derived metabolite preparation.”

Clinical evidence for metabolite‐based approaches: Clinical trials exploring the effects of fermentation‐derived preparations in metabolic syndrome and IBD are underway. Notably, a recent randomized placebo‐controlled trial demonstrated that oral administration of a fermentation‐derived postbiotic (containing complex microbial metabolites alongside inactivated microbial biomass) during antibiotic therapy significantly increased gut microbiome diversity (+40%) and enriched health‐associated taxa, including Lachnospiraceae (Schluter et al. 2026). While promising, this study used a preparation that contained inactivated microbial biomass and thus met the ISAPP definition of a postbiotic. For metabolite‐only preparations (purified SCFAs or cell‐free supernatants without microbial components), human clinical trial evidence remains limited.

Regulatory considerations: The regulatory pathway for metabolite‐based preparations depends on whether they contain microbial biomass:

- Postbiotics (containing inactivated microbial cells) may be classified as foods, supplements, or drugs depending on the jurisdiction and health claims made (Sanders et al. 2019).
- Purified metabolites (e.g., butyric acid and propionic acid) are typically regulated as food ingredients or chemical substances, with regulatory requirements depending on their history of safe use and the intended application (Sanders et al. 2019).
- The regulatory landscape is not uniform globally; evidentiary requirements for health claims are substantial regardless of classification (Salminen et al. 2021; Sanders et al. 2019).

The ISAPP consensus panel explicitly listed purified microbial metabolites (e.g., organic acids) and filtrates without cell components as not falling within the postbiotic definition. Therefore, researchers and product developers should exercise terminological precision: preparations containing inactivated microbial cells qualify as postbiotics; purified metabolites or cell‐free supernatants without biomass should be termed “metabolite‐based preparations” or described by their chemical constituents.

## 6 Challenges, Remaining Knowledge Gaps, and a Research Priority Framework

Despite significant progress in understanding the immunomodulatory and metabolic functions of Lachnospiraceae in preclinical models, translating these findings into clinical and food applications faces substantial obstacles. A sober assessment of the current evidence reveals that the gap between mechanistic insights and practical applications remains wide. Below we define five priority research areas that must be addressed before any therapeutic or food application can be realistically considered. In contrast to a speculative roadmap with fixed timelines, we present a framework organized by the prerequisites that must be met at each stage of translation. The time required to achieve these prerequisites is inherently uncertain and depends on progress across multiple scientific, technological, and regulatory fronts (Figure 5).

### 6.1 General Limitations and Methodological Considerations

Several cross‐cutting limitations affect the entire evidence base for Lachnospiraceae research, irrespective of the clinical domain.

- Causality and attribution: The predominant limitation across all fields is the inability to distinguish correlation from causation. Nearly all human data are cross‐sectional or retrospective, and positive correlations between Lachnospiraceae abundance and clinical outcomes cannot establish whether these bacteria drive effects or merely reflect a healthy microbiome. Confounders—diet, medication, and disease duration—are seldom adequately controlled.
- Strain‐specificity and generalizability: Functional heterogeneity across genera and strains invalidates any extrapolation from A. soehngenii (the only strain with RCT and GRAS data) to other family members. Each strain requires independent evaluation.
- Safety and regulatory gaps: Safety data are limited to short‐term trials in immunocompetent adults. Risks in immunocompromised populations are unknown; regulatory recognition (A. soehngenii GRAS) applies only to food use.
- Methodological limitations: Reliance on fecal SCFA as a proxy for tissue‐level activity is problematic, as fecal levels reflect unabsorbed fractions. 16S rRNA sequencing provides only genus‐level resolution, insufficient for strain‐specific functional attribution.
- Publication bias: Positive findings are preferentially published, likely overestimating effect sizes. Null or negative results are underrepresented.

### 6.2 Strain‐Level Characterization (From Family‐Level Correlation to Strain‐Level Causality)

Problem: Most studies pool family‐ or genus‐level data, ignoring vast functional heterogeneity that exists within genera. It is established that metabolic and immunomodulatory capacities are strain‐specific—two strains of the same species can differ in butyrate production, adhesion properties, and immunomodulatory effects by orders of magnitude. Generalizations about “Lachnospiraceae” as a homogeneous family are scientifically unjustified and have likely contributed to the overinterpretation of correlational findings.

Research Priority:

- Large‐scale culturomics to isolate hundreds of Lachnospiraceae strains from diverse human populations (different geographical regions, ages, and health states). This is resource‐intensive and has been accomplished for only a few genera to date (Sorbara et al. 2020; Plomp et al. 2024).
- Whole‐genome sequencing and comparative genomics to identify strain‐specific functional gene clusters (e.g., butyrate synthesis operons, adhesion factors, and antibiotic resistance genes) (Lin et al. 2024).
- High‐throughput in vitro functional screening using immune cell co‐culture (Treg/Th17 differentiation assays), gut‐on‐a‐chip models, and Caco‐2 barrier assays (Bhutta et al. 2024).
- Development of CRISPR‐Cas9‐based gene editing tools for Lachnospiraceae to validate candidate immunomodulatory genes. This is currently lacking for most genera and represents a major technical bottleneck (Ma et al. 2025).
- Prioritize genera with the highest translational potential (Roseburia, Eubacterium, Anaerostipes, and Blautia), while also exploring understudied genera for novel functions. A critical caveat is that findings from one genus should not be extrapolated to others without direct experimental validation (Lin et al. 2024).

### 6.3 Multi‐Omics Integration (From Taxonomy to Functionality)

Research Priority: Combine multiple omics layers in the same cohort:

- Metagenomics (strain‐level resolution and functional gene prediction) (Lin et al. 2024).
- Metabolomicsm (targeted SCFA, bile acid, and tryptophan metabolite quantification) (Belenguer et al. 2006).
- Host transcriptomics (single‐cell RNA sequencing of intestinal immune cells from biopsies) (Bhutta et al. 2024).
- Use machine learning (random forest, gradient boosting, and neural networks) to build predictive models of intervention response (Boodaghidizaji et al. 2025). For example, the hypothesis that baseline *Roseburia* abundance >3% may predict responsiveness to fiber‐based interventions requires prospective testing. To date, this remains a hypothesis, not an established clinical tool (Aslam et al. 2026).

### 6.4 Integrated Research Priority Framework

On the basis of the critical appraisal across all disease contexts, the following integrated priorities emerge:

- Causality: Conduct multicenter prospective cohorts with pre‐disease baseline sampling and rigorous confounder adjustment.
- Strain‐level resolution: Isolate and genomically characterize diverse Lachnospiraceae strains; develop high‐throughput functional screening platforms.
- RCTs with defined strains: The single most important gap—conduct RCTs testing defined strains in patient populations with disease‐relevant clinical endpoints.
- Safety monitoring: Long‐term safety data in diverse populations, including immunocompromised individuals, are essential.
- Food matrix research: Systematic studies on processing effects, matrix interactions, and dose–response relationships.
- Causal inference methods: Employ Mendelian randomization, longitudinal sampling, and appropriate control groups.

### 6.5 Articulating the Translational Barriers: A Critical Synthesis

The literature often presents the translational pathway for Lachnospiraceae as an exciting frontier. However, a critical examination reveals four fundamental barriers that are frequently understated.

#### 6.5.1 Barrier 1: Strain‐Level Heterogeneity and Functional Unpredictability

Functional heterogeneity within the Lachnospiraceae family is substantial. Even strains of the same species can differ in SCFA profiles, immunomodulatory effects, adhesion capacity, oxygen tolerance, and the presence of virulence or antibiotic resistance genes. Consequently, observational studies at the family or genus level are of limited utility for strain selection. Without strain‐level resolution, it is impossible to predict whether a given strain will be beneficial, neutral, or harmful. A “one strain fits all” strategy is highly unlikely to succeed.

#### 6.5.2 Barrier 2: Scarcity of Robust Clinical Evidence

With the sole exception of A. soehngenii (moderate evidence for glycemic outcomes), no RCT has tested a defined Lachnospiraceae strain in any disease. The clinical evidence base for all other strains consists of small uncontrolled pilot studies (*n* = 10–30), mixed probiotic formulations where effects cannot be attributed to Lachnospiraceae, observational studies that cannot establish causality, and short‐term studies (4–12 weeks) with surrogate endpoints. Claims about therapeutic efficacy remain premature; the field is still in the hypothesis‐generation phase.

#### 6.5.3 Barrier 3: Substantial Regulatory Complexity

The regulatory landscape for live microorganisms is fragmented and contingent on strain, intended use, and jurisdiction:

- GRAS (US): Applies to food ingredients, not therapeutic claims. Only A. soehngenii CH106 has received a “no questions” GRAS response (GRN No. 1065, April 2023), strictly for food use. No other strain has GRAS status.
- QPS (European Union [EU]): No Lachnospiraceae strain—including A. soehngenii—has achieved QPS status.
- Novel Food (EU): Requires comprehensive safety assessment for foods without significant consumption history.
- LBPs (FDA/European Medicines Agency [EMA]): Requires full biologic drug development pathway (IND, Phases 1–3 trials, biologics license application [BLA]/marketing authorization application [MAA]) for therapeutic use.

A strain that is GRAS for food is not automatically approvable as a therapeutic; the A. soehngenii case exemplifies regulatory complexity, not simplicity.

#### 6.5.4 Barrier 4: Technological Constraints for Food Applications

Lachnospiraceae are generally obligate anaerobes, posing unique challenges:

- Cultivation: Requires anaerobic conditions, specialized media, and oxygen‐free processing, increasing costs and limiting facilities.
- Viability: Limited tolerance to oxygen, acidic pH, and bile acids; viability declines rapidly during storage and gastrointestinal transit.
- Formulation: Encapsulation technologies are being explored but have not been optimized for Lachnospiraceae; oxygen‐scavenging packaging adds cost.
- Shelf‐life: Products containing live anaerobes typically last weeks to a few months under refrigeration, limiting distribution.

Even if a therapeutically effective strain is identified, translation into a commercially viable food product is nontrivial; technological challenges may be as difficult to overcome as biological and clinical ones.

### 6.6 Personalized Intervention Strategies—A Long‐Term Research Horizon

Research Priority: Stratify patients based on

- Baseline microbiome composition (e.g., abundance of Roseburia, E. rectale, and primary degraders such as Bifidobacterium).
- Host genetic background (e.g., human leukocyte antigen [HLA]‐DQ2/DQ8, NOD2/CARD15, IL‐10 promoter single nucleotide polymorphisms [SNPs]).
- Metabolic profile (e.g., insulin sensitivity and inflammatory markers).
- Dietary patterns and fiber intake.
- Use adaptive trial designs where interventions are modified based on real‐time microbiome feedback (e.g., if a patient's baseline Roseburia is low, provide a high‐inulin intervention; if already high, use a maintenance fiber mixture).

Critical caveat: This precision approach is not ready for clinical practice. It represents a long‐term research horizon that requires:

- Validation of predictive biomarkers in independent cohorts.
- Development of rapid, inexpensive, and clinically deployable microbiome profiling tools.
- Demonstration in prospective trials that biomarker‐guided stratification improves outcomes compared with a one‐size‐fits‐all approach.

The substantial inter‐individual variability in response to dietary interventions is well documented, and personalized strategies are conceptually attractive. However, the evidence base to support clinical implementation is currently absent.

### 6.7 Summary: A Realistic Perspective on the Translational Pathway

The enthusiasm for Lachnospiraceae must be tempered by recognition of the evidence gaps and translational barriers.

Key points:

- Preclinical mechanisms are well characterized, but their translation to humans is unproven.
- Correlational human studies are abundant, but causal evidence is lacking.
- Regulatory approvals for Lachnospiraceae‐based products are rare; to date, only A. soehngenii CH106 (food use only); no strain has QPS or LBP approval.
- Technological challenges for food applications (anaerobic cultivation, viability, formulation, and shelf‐life) are substantial and often underestimated.
- Personalized approaches are conceptually promising but lack clinical validation.

A responsible interpretation of the current evidence is that the field is at an early, exploratory stage. Although the biological plausibility is strong, the path to clinical and food applications is long and uncertain. Rigorous research addressing the prerequisites outlined above—not speculation about timelines—will ultimately determine whether and how Lachnospiraceae can be translated into tangible benefits for human health.

## 7 Conclusion—A Realistic Appraisal of Evidence, Knowledge Gaps, and Research Priorities

The substantial body of research accumulated over the past two decades has established Lachnospiraceae as abundant, fiber‐fermenting constituents of the human gut microbiota that produce immunomodulatory SCFAs and maintain gut barrier integrity in preclinical models. However, a sober assessment of the evidence reveals a persistent and substantial gap between mechanistic plausibility and demonstrated clinical utility. This conclusion synthesizes the key findings of this review, articulates the principal knowledge gaps that remain unaddressed, and proposes a realistic framework for future research priorities.

### 7.1 The Evidence Gap: From Correlation to Causation

The single most critical knowledge gap is causality. Despite extensive correlational evidence linking Lachnospiraceae abundance to autoimmune diseases, metabolic disorders, and cancer immunotherapy response, no randomized controlled trial has demonstrated that increasing Lachnospiraceae abundance or supplementing with specific strains improves clinical outcomes in humans—with the sole exception of A. soehngenii in glycemic control (moderate evidence). For all other strains and indications, the evidence remains low to very low. Even for A. soehngenii, the evidence is limited to metabolic disease populations and does not establish efficacy for other indications. The field remains predominantly in the hypothesis‐generation phase, not the evidence‐based application phase.

Several foundational constraints critically temper translational inference. First, the predominance of cross‐sectional and retrospective human data renders causality unattainable, as observed associations offer no clarity on whether changes in Lachnospiraceae abundance drive disease or merely reflect it. Second, considerable strain‐level heterogeneity invalidates any generalization from higher taxonomic levels to functionally distinct strains. Third, with the exception of A. soehngenii (moderate evidence in metabolic disease), the complete absence of published RCTs employing defined Lachnospiraceae strains means that therapeutic efficacy for all other strains remains speculative (very low evidence). Fourth, although FMT studies have yielded preliminary signals, their complex microbial mixtures preclude isolating Lachnospiraceae‐specific effects from those of co‐transplanted taxa. Finally, safety assessments are restricted to short‐term, small‐scale cohorts, leaving substantial uncertainties about long‐term tolerability and risks in immunocompromised individuals.

These limitations are not merely technical—they are fundamental. Until they are addressed through rigorous, hypothesis‐driven research—prioritizing causality, strain‐level specificity, RCTs with defined strains, and long‐term safety evaluation—claims of therapeutic efficacy remain premature. The current literature is predominantly hypothesis‐generating and falls far short of providing the robust evidence needed for clinical or therapeutic application.

### 7.2 Safety Considerations—A Balanced Perspective

The safety profile of Lachnospiraceae‐based products must be considered on a strain‐specific basis, and generalizations about the family as a whole are not justified. Although defined nonpathogenic strains, such as A. soehngenii CH106, have received GRAS status for food use and have shown no serious adverse events in short‐term RCTs (Ilias et al. 2025), safety considerations must be approached with appropriate caution:

- Case reports of bacteremia involving Lachnospiraceae members—including Lachnoanaerobaculum gingivalis in an immunocompromised patient (Okada et al. 2022) and Extibacter muris in an immunocompetent patient (Gourdel et al. 2025)—demonstrate that these organisms can cause bloodstream infections under certain conditions. The incidence rate of such infections is unknown, and population‐level epidemiological data are not available.
- Long‐term safety data for Lachnospiraceae supplementation in humans are lacking; most RCTs have follow‐up periods of 4–12 weeks, insufficient to assess rare or chronic adverse events.
- Safety in immunocompromised populations—including cancer patients, transplant recipients, and those with primary immunodeficiencies—has not been systematically evaluated. These populations may be at elevated risk for translocation and bacteremia.
- Regulatory approvals for Lachnospiraceae‐based products are rare. To date, only A. soehngenii CH106 has achieved GRAS status in the US, and this applies strictly to food use. No Lachnospiraceae strain—including *A. soehngenii*—has received QPS status in the EU, and none has regulatory approval as an LBP for any therapeutic indication (Baydoun et al. 2025)

A balanced interpretation of the current evidence is that Lachnospiraceae appear to be safe for food use when using defined, well‐characterized nonpathogenic strains, but this does not generalize to all strains or all applications. Strain‐specific safety assessment remains essential, and therapeutic applications require substantially more evidence.

### 7.3 Regulatory and Technological Realities

The translation of Lachnospiraceae‐based products from research to market is constrained by substantial regulatory and technological realities that are often understated in the literature:

- Regulatory uncertainty: The FDA's proposed rule (Regulation Identification Number [RIN] 0910‐AJ02) that would mandate premarket notification for GRAS substances is anticipated for publication in December 2026, with a final rule unlikely before late 2028 or 2029 at the earliest (https://www.lexology.com/library/detail.aspx?g=ae1d08d1‐38d4‐45bb‐9abb‐99b0b21c5447, https://www.venable.com/insights/publications/2026/07/new‐details‐emerge‐on‐fdas‐gras‐rulemaking). This means that the regulatory framework for GRAS substances—including Lachnospiraceae strains—is currently in flux.
- Regulatory fragmentation: The pathway for a product depends on its intended use—food, supplement, or therapeutic—and the jurisdiction. GRAS (US, https://www.fda.gov/food/food‐ingredients‐packaging/generally‐recognized‐safe‐gras), QPS (qualified‐presumption‐safety, EU, https://www.efsa.europa.eu/en/topics/topic/qualified‐presumption‐safety‐qps), Novel Food (EU, https://food.ec.europa.eu/food‐safety/novel‐food_en), and LBP (live‐biotherapeutic‐products, FDA/EMA, https://www.edqm.eu/en/w/live‐biotherapeutic‐products‐lbps‐european‐pharmacopoeia‐commission‐sets‐unprecedented‐quality‐requirements) pathways have fundamentally different evidentiary requirements, timelines, and assessment levels (EFSA Panel on Biological Hazards (BIOHAZ) et al. 2026; Baydoun et al. 2025.
- Technological challenges: Lachnospiraceae are strict anaerobes, and their viability during processing, storage, and gastrointestinal transit is a significant challenge. Although formulation strategies, such as polymeric encapsulation, have shown promise for model anaerobes, these findings do not generalize to all strains and require strain‐specific optimization.
- Strain‐specificity imperative: The functional heterogeneity within the Lachnospiraceae family is substantial. Findings from one strain—including A. soehngenii—cannot be extrapolated to others without direct experimental validation.

### 7.4 Integrated Research Priorities

Rather than proposing “consensus take‐home messages” that imply a level of certainty that the evidence does not support, we present the following as integrated research priorities that reflect the current state of knowledge and the principal knowledge gaps:

Priority 1: Establish Causality Through Longitudinal and Interventional Studies

- Conduct multicenter prospective cohorts (*n* ≥ 200 per disease, ≥12 months follow‐up) with repeated sampling to move beyond cross‐sectional correlations.
- Use Mendelian randomization and causal mediation analysis to infer causality where RCTs are not feasible.
- Critical caveat: Even well‐designed prospective studies can only strengthen causal inference, not definitively prove causation—that requires RCTs.

Priority 2: Achieve Strain‐Level Resolution

- Isolate and genomically characterize hundreds of Lachnospiraceae strains from diverse human populations.
- Develop high‐throughput functional screening platforms to identify strain‐specific immunomodulatory and metabolic capacities.
- Critical caveat: Functional heterogeneity is substantial; family‐ or genus‐level generalizations are scientifically unjustified.

Priority 3: Conduct Rigorous RCTs with Defined Strains

- The single most important evidence gap is the absence of RCTs testing defined Lachnospiraceae strains in patient populations.
- Use disease‐relevant clinical endpoints (not just surrogate biomarkers).
- Include long‐term safety follow‐up, particularly in immunocompromised populations.

Priority 4: Address Regulatory and Technological Barriers

- Develop standardized, validated methods for strain characterization, viability assessment, and stability testing.
- Engage with regulatory agencies early in product development to understand the evidentiary requirements for the intended use.
- Critical caveat: Regulatory pathways are fragmented, uncertain, and contingent on intended use—not simply on the microorganism itself.

Priority 5: Translate Mechanistic Insights into Evidence‐Based Dietary Guidance

- Design fiber‐rich, minimally processed foods that selectively enrich butyrate‐producing gut bacteria in the general population. Evidence‐based strategies include:
  - Using whole grains rather than refined flours
  - Incorporating resistant starch (e.g., cooked‐then‐cooled potatoes, rice, or pasta; green bananas)
  - Including pectin‐rich fruits (apples, citrus, and plums)
  - Avoiding over‐processing that destroys fiber structure (e.g., fine milling and high‐shear extrusion)

Critical caveat: These dietary strategies are safe, inexpensive, and scalable—and they do not require regulatory approval for live bacteria. However, the specific contribution of Lachnospiraceae to the observed benefits remains unproven. High‐fiber diets are recommended for general health, but the attribution of benefits specifically to Lachnospiraceae—rather than to other SCFA producers such as Faecalibacterium—is not yet justified.

### 7.5 A Realistic Perspective on Clinical Translation

The question of when Lachnospiraceae‐based interventions may become clinically available cannot be answered with a specific timeline. The timeline depends on progress across multiple fronts:

- Biological: Identifying which specific strains (or consortia) confer therapeutic benefits
- Clinical: Conducting rigorous RCTs with disease‐relevant endpoints.
- Technological: Scaling up production of strict anaerobes under GMP conditions while maintaining viability and stability.
- Regulatory: Navigating fragmented and evolving regulatory frameworks across jurisdictions.

A responsible assessment is that the field is at an early, exploratory stage. Although the biological plausibility is strong, the path to clinical and food applications is long and uncertain. The “within the next decade” prediction is speculative and does not reflect the substantial barriers that remain. Progress will be driven by rigorous research addressing the priorities outlined above—not by timelines or optimism.

### 7.6 Final Synthesis

In summary, although Lachnospiraceae represent a scientifically compelling family of gut bacteria with well‐characterized immunomodulatory mechanisms in preclinical models, their translation to clinical and food applications faces substantial barriers: The evidence landscape for Lachnospiraceae (see Section 5.8 for detailed discussion of A. soehngenii, the only strain with RCT data) is defined by a stark contrast between preclinical promise and clinical uncertainty.

The knowledge gained from studying Lachnospiraceae has already informed dietary guidelines that promote gut health through high‐fiber diets. However, Lachnospiraceae‐based probiotics, postbiotics, or LBPs are not yet ready for widespread clinical use. Future research must prioritize causality, strain‐level specificity, rigorous clinical testing, and food‐focused translation. With continued progress—and acknowledging the substantial barriers that remain—Lachnospiraceae‐based or Lachnospiraceae‐inspired interventions may eventually become valuable tools for managing immune‐mediated and metabolic diseases. The timeline for achieving this goal remains inherently uncertain.

## Author Contributions

**Ruijun Wang**: formal analysis, methodology, writing – original draft, writing – review and editing, funding acquisition. **Zhiqi Li**: formal analysis, methodology, software, writing – review and editing. **Lina Zhang**: supervision, formal analysis, methodology, writing – review and editing, visualization. **Yuanming Pan**: conceptualization, resources, supervision, funding acquisition, writing – review and editing. **Zhanbiao He**: writing – original draft, investigation, methodology, funding acquisition.

## Funding

Financial support was provided by Inner Mongolia Autonomous Region Clinical Medicine Research Center of Nervous System Diseases, Hohhot Religion High‐quality Developmental and Advantageous Key Clinical Project of Neurological System Disease, National Natural Science Foundation of China (82060906), and Key project of Inner Mongolia Medical University (YKD2022ZD002); Natural Science Foundation of Inner Mongolia Autonomous Region (2020MS08146); Inner Mongolia Autonomous Region Health Commission Medical Hygiene Science Project (202201307); 2024 Science and Technology Project for Building High‐level Clinical Specialties in Capital Region Public Hospitals (2024SGGZ070); 2024 Central‐Guided Local Science and Technology Development Funding Project (2024ZY0086) to Ruijun Wang. Beijing Traditional Chinese Medicine Science and Technology Development Fund Project (BJZYYB‐2025‐24), Beijing Municipal Public Welfare Development and Reform Pilot Project for Medical Research Institutes (JYY2023‐14) and the Research Project of Inner Mongolia Medical University Affiliated Hospital (2023NYFYLHZD007), Beijing Municipal Administration of Hospital Incubating Program (PX2023059); The Capability Program of Beijing Tuberculosis & Thoracic Tumor Research Institute (NLTS2024‐16) to Yuanming Pan. The General Program of Inner Mongolia Medical University (YDK2025MS017) to Zhanbiao He.

## Ethics Statement

This study did not involve human or animal subjects, and thus, no ethical approval was required. The study protocol adhered to the guidelines established by the journal.

## Consent

The authors have nothing to report.

## Conflicts of Interest

The authors declare no conflicts of interest.

## Data Availability

Data sharing is not applicable to this article as no datasets were generated or analyzed during the current study.
